# Targeting the lipid kinase PIKfyve upregulates surface expression of MHC class I to augment cancer immunotherapy

**DOI:** 10.1073/pnas.2314416120

**Published:** 2023-11-27

**Authors:** Yi Bao, Yuanyuan Qiao, Jae Eun Choi, Yuping Zhang, Rahul Mannan, Caleb Cheng, Tongchen He, Yang Zheng, Jiali Yu, Mahnoor Gondal, Gabriel Cruz, Sara Grove, Xuhong Cao, Fengyun Su, Rui Wang, Yu Chang, Ilona Kryczek, Marcin Cieslik, Michael D. Green, Weiping Zou, Arul M. Chinnaiyan

**Affiliations:** ^a^Michigan Center for Translational Pathology, University of Michigan, Ann Arbor, MI 48109; ^b^Department of Pathology, University of Michigan, Ann Arbor, MI 48109; ^c^Rogel Cancer Center, University of Michigan, Ann Arbor, MI 48109; ^d^Department of Surgery, University of Michigan, Ann Arbor, MI 48109; ^e^Center of Excellence for Cancer Immunology and Immunotherapy, University of Michigan, Ann Arbor, MI 48109; ^f^Department of Computational Medicine and Bioinformatics, University of Michigan, Ann Arbor, MI 48109; ^g^Department of Radiation Oncology, University of Michigan, Ann Arbor, MI 48109; ^h^Veterans Affairs Ann Arbor Healthcare System, Ann Arbor, MI 48109; ^i^HHMI, University of Michigan, Ann Arbor, MI 48109; ^j^Department of Urology, University of Michigan, Ann Arbor, MI 48109

**Keywords:** PIKfyve, MHC class I, immunotherapy, cancer

## Abstract

Despite the remarkable clinical success of immunotherapies in a subset of cancer patients, many fail to respond to treatment and exhibit resistance. Here, we found that genetic or pharmacologic inhibition of the lipid kinase PIKfyve, a regulator of autophagic flux and lysosomal biogenesis, upregulated surface expression of major histocompatibility complex class I (MHC-I) in cancer cells via impairing autophagic flux, resulting in enhanced cancer cell killing mediated by CD8^+^ T cells. Genetic depletion or pharmacologic inhibition of PIKfyve elevated tumor-specific MHC-I surface expression, increased intratumoral functional CD8^+^ T cells, and slowed tumor progression in multiple syngeneic mouse models. Importantly, enhanced antitumor responses by *Pikfyve*-depletion were CD8^+^ T cell- and MHC-I-dependent, as CD8^+^ T cell depletion or *B2m* knockout rescued tumor growth. Furthermore, PIKfyve inhibition improved response to immune checkpoint blockade (ICB), adoptive cell therapy, and a therapeutic vaccine. High expression of *PIKFYVE* was also predictive of poor response to ICB and prognostic of poor survival in ICB-treated cohorts. Collectively, our findings show that targeting PIKfyve enhances immunotherapies by elevating surface expression of MHC-I in cancer cells, and PIKfyve inhibitors have potential as agents to increase immunotherapy response in cancer patients.

## Significance

Immunotherapies have shown clinical success in certain indications, but many patients fail to respond to treatment and exhibit resistance. Despite tremendous efforts to solve this clinical challenge, developing therapeutic strategies to improve response to immunotherapies remains a pressing clinical problem. The lack of a thorough understanding of the molecular processes that cancer cells adopt to downregulate MHC-I (major histocompatibility complex class I) to evade antitumor immunity has hindered the development of a rational combination treatment for overcoming resistance to immunotherapies. This study identifies therapeutic targeting of PIKfyve as a strategy to upregulate surface expression of MHC-I in cancer cells and thus enhance response to immunotherapies across various cancer types, including those such as pancreatic cancer that are often unresponsive to immunotherapies.

Immunotherapy is a type of treatment that harnesses the power of the immune system to fight diseases, particularly cancer (1–4). Many patients, however, fail to benefit from immunotherapies by exhibiting primary resistance (5–8). Understanding what enables successful immunotherapy treatment and developing strategies that overcome resistance to immunotherapies remains a critical pursuit (5–8).

The role of CD8^+^ T cells in anticancer immunity and the clinical success of immunotherapies has been well established (9–12). For example, antigen presentation by major histocompatibility complex class I (MHC-I) on tumor cells is crucial for CD8^+^ T cells to recognize malignant cells (13–15). While complete genetic loss of MHC-I components is rare (14), malignant cells often down-regulate expression of MHC-I, leading to escape from CD8^+^ T cell recognition and failure of immunotherapies (13, 14, 16–19).

Autophagy has recently been found to degrade MHC-I, resulting in decreased MHC-I surface expression in pancreatic ductal adenocarcinoma (PDAC) (17). Inhibition of autophagy restores tumor-specific MHC-I surface expression, leading to increased intratumoral CD8^+^ T cells and immune checkpoint blockade (ICB) sensitization in mouse PDAC models (17). Concordantly, accumulating evidence suggests that autophagy inhibition may augment antitumor immunity and enhance efficacy of ICB therapy (20–22). However, autophagy inhibitors tested in the clinic, chloroquine and hydroxychloroquine, show limited cellular uptake in acidic conditions (23). This is potentially attributed to the low clinical efficacy of these autophagy inhibitors (24–26), as the tumor microenvironment is acidic (27). Furthermore, chloroquine and hydroxychloroquine are non-specific autophagy inhibitors (28, 29). Therefore, novel therapeutic strategies are needed to target autophagy.

PIKfyve is a class III lipid kinase that phosphorylates phosphatidylinositol 3-phosphate to generate PI(3, 5)P2 (phosphatidylinositol 3,5-biphosphate). PIKfyve, which is related to the more well-known lipid-related kinase PIK3CA, plays an essential role in autophagy and lysosomal adaptation processes. PIKfyve inhibition has been shown to mediate autophagy dysfunction by blocking autophagic flux and to exhibit antitumor efficacy in various cancer types (30–33). At least two PIKfyve inhibitors, apilimod and ESK981 (formerly CEP-11981), are in clinical development for cancer therapy (34–36). However, whether and how PIKfyve serves as a target for upregulating tumor-specific MHC-I surface expression and whether apilimod or ESK981 treatment augments CD8^+^ T cell–dependent immunotherapies remain to be explored.

Here, we found that genetic depletion or pharmacologic inhibition of PIKfyve upregulated surface expression of MHC-I in cancer cells, leading to enhanced antigen presentation and improved CD8^+^ T cell–mediated cancer cell killing in vitro. Concordantly, genetic or pharmacologic inhibition of PIKfyve upregulated tumor-specific MHC-I surface expression, increased intratumoral functional CD8+ T cells, and retarded tumor progression in syngeneic mouse models. Finally, we tested the combination of PIKfyve inhibitor and various CD8^+^ T cell–dependent immunotherapies, including ICB, adoptive cell therapy (ACT), and a therapeutic vaccine, and found that PIKfyve inhibition markedly enhanced efficacy of these immunotherapies in pre-clinical models. Collectively, our findings suggest that targeting PIKfyve may enhance CD8^+^ T cell–dependent immunotherapies via elevating surface expression of MHC-I in cancer cells.

## Results

### Genetic or Pharmacologic Inhibition of PIKfyve Induces MHC-I Surface Expression.

**Fig. 1.**
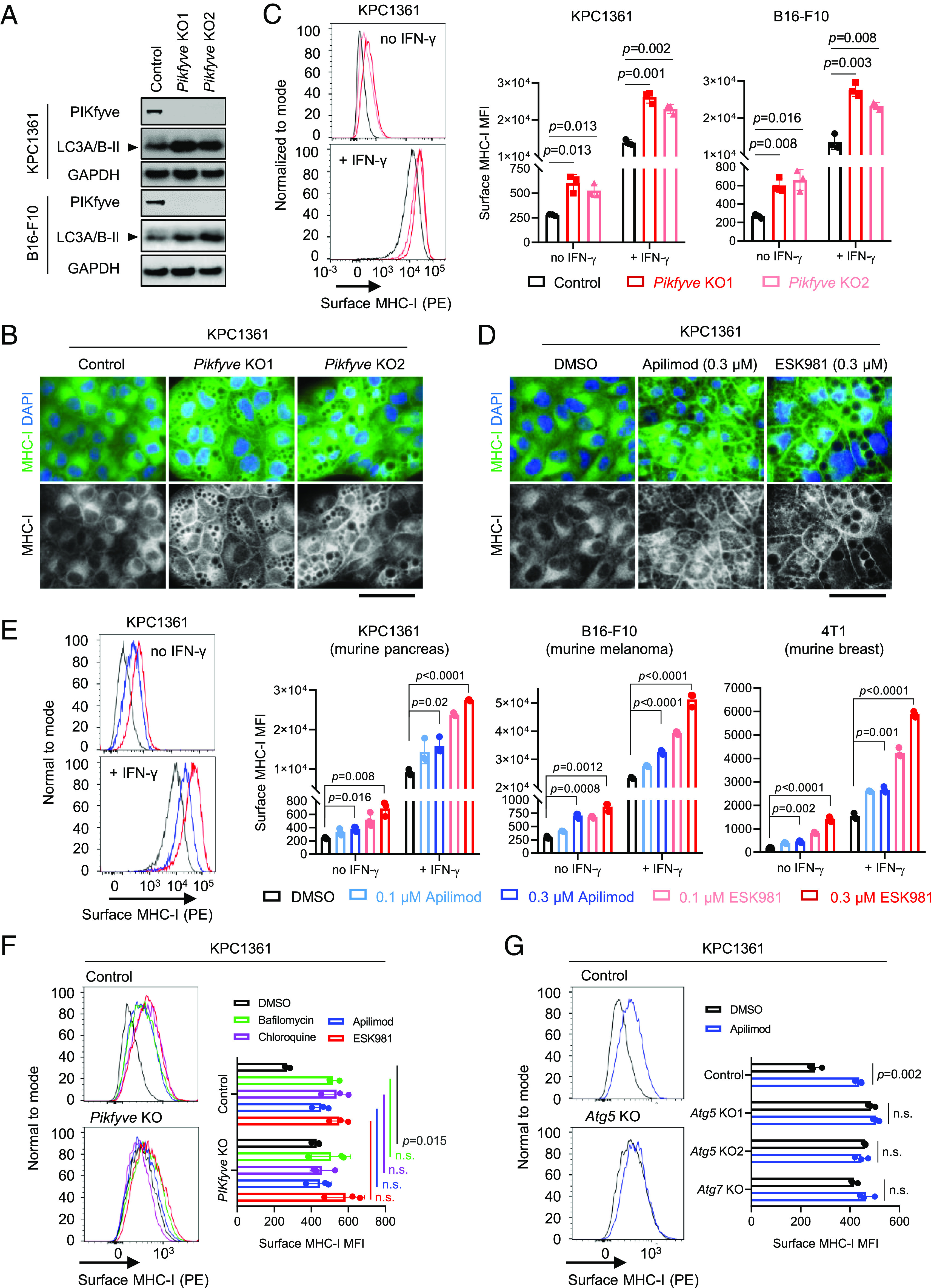
Genetic or pharmacologic inhibition of PIKfyve induces MHC-I surface expression. (*A*) Immunoblot analysis assessing levels of the indicated proteins in KPC1361 and B16-F10. Images are representative of two independent biological replicates. (*B*) Immunofluorescence assessing MHC-I localization in KPC1361 with non-targeting single-guide RNA (control), or independent single-guide RNAs depleting *Pikfyve* (*Pikfyve* KO1 and *Pikfyve* KO2), stimulated with IFN-γ at 10 ng/mL for 24 h. Images are representative of two independent biological replicates. (Scale bar: 50 µm.) (*C*) Representative traces (*Left*) and quantification (*Right*) of flow cytometry measuring MHC-I surface expression in control or *Pikfyve* KO cells, treated with or without IFN-γ at 10 ng/mL for 24 h. (*D*) Immunofluorescence assessing MHC-I localization in KPC1361 treated with the indicated agents at the indicated concentrations for 24 h and stimulated with IFN-γ at 10 ng/mL for 24 h. Images are representative of two independent biological replicates. (Scale bar: 50 µm.) (*E*) Representative images (*Left*) and quantification (*Right*) of flow cytometry measuring MHC-I surface expression in the indicated cells stimulated with or without IFN-γ at 10 ng/mL and treated with the agents at the indicated concentrations for 24 h. (*F*) Representative images (*Left*) and quantification (*Right*) of flow cytometry measuring MHC-I surface expression in KPC1361 with or without *Pikfyve* KO, following treatment of the indicated agents for 24 h. Concentrations of the treatments were 200 nM for bafilomycin, 30 μM for chloroquine, 300 nM for apilimod, and 100 nM for ESK981. (*G*) Representative images (*Left*) and quantification (*Right*) of flow cytometry measuring MHC-I surface expression in KPC1361 with non-targeting control, or single-guide RNAs depleting *Atg5* or *Atg7*, following treatment of 300 nM apilimod for 24 h. *Atg5* KO1 and KO2 were generated with independent single-guide RNAs. Data in (*C*, *E*, and *F*) were acquired with three independent biological replicates, presented as mean ± SD. Data in (*G*) were acquired with technical triplicates representative of two independent biological replicates. All statistics were acquired by two-tailed Student’s *t* test with Bonferroni correction. MFI: mean fluorescence intensity. n.s.: not significant.

To examine whether targeting PIKfyve induces MHC-I surface expression, we first knocked out *Pikfyve* in different murine cancer cell models—KPC1361 derived from a pancreatic tumor of a genetically engineered mouse model (*LSL-Kras^G12D/+^; LSL-Trp53^R172H/+^; Pdx1-Cre*) and the melanoma model B16-F10 (Fig. 1*A*). Consistent with previous reports (30, 32), *Pikfyve* depletion led to accumulation of the lipidated form (LC3A/B-II) of MAP1LC3A/B protein (LC3A/B) (Fig. 1*A*) and formation of vacuoles in vitro in the cancer cells (Fig. 1*B*). Employing immunofluorescence, we found that knockout of *Pikfyve* resulted in increased cell surface expression of MHC-I (Fig. 1*B*). To quantify the surface expression of MHC-I, we further employed flow cytometry analysis and confirmed that *Pikfyve* depletion elevated both constitutive and IFN-γ-induced surface expression of MHC-I (Fig. 1*C*). We then sought to examine these effects with two phase I-cleared orally bioavailable PIKfyve inhibitors, apilimod and ESK981. Apilimod or ESK981 treatment also increased cell surface expression of MHC-I (Fig. 1*D*), and upregulated both constitutive and induced surface expression of MHC-I in various murine cancer types, including KPC1361 (pancreas), B16-F10 (melanoma), 4T1 (breast), and MyC-CaP (prostate), in a dose-dependent manner (Fig. 1*E* and *SI Appendix*, Fig. S1*A*). Importantly, the increased MHC-I surface expression by PIKfyve inhibition was also observed in various human cancer models, including MIA PaCa2, LNCaP, and prostate cancer patient–derived xenografts (*SI Appendix*, Fig. S1 *B*–*D*). In line with previous reports (30, 32), treatment with the PIKfyve inhibitors also led to LC3A/B-II accumulation (*SI Appendix*, Fig. S1*E*) and vacuole formation (Fig. 1*D*). PIKfyve has been shown to regulate the formation of autolysosomes and, thus, protein degradation (31, 32). We observed that total protein levels, but not mRNA levels, of MHC-I were upregulated by PIKfyve inhibition (*SI Appendix*, Fig. S1 *E* and *F*). To evaluate whether the action of PIKfyve inhibitors was on-target, we treated *Pikfyve*-wild-type or *Pikfyve*-null cancer cells with apilimod or ESK981 and found that upregulation of MHC-I surface expression by PIKfyve inhibition was only observed in the wild-type cells but not *Pikfyve*-null cells (Fig. 1*F*). These data confirmed that the action of PIKfyve inhibitors was on-target.

PIKfyve inhibition has previously been shown to impair autophagy (30, 32). We thus sought to determine whether inhibition of PIKfyve elevated MHC-I surface expression by perturbing autophagic flux. We disrupted autophagy with bafilomycin or chloroquine in *Pikfyve*-wild-type or *Pikfyve*-null cancer cells. In line with what has been reported (17), we found that inhibition of autophagy by bafilomycin or chloroquine induced MHC-I surface expression in the *Pikfyve*-wild-type cells (Fig. 1*F*). While knockout of *Pikfyve* alone elevated MHC-I surface expression, it failed to further upregulate the levels of MHC-I surface expression upon autophagy inhibition (Fig. 1*F*). These data indicate that the elevated MHC-I surface expression by PIKfyve inhibition was achieved via autophagy impairment. Moreover, we disrupted autophagy with a genetic approach by depleting *Atg5* or *Atg7*. Successful knockout of *Atg5* or *Atg7* was indicated by complete loss of ATG5 or ATG7 protein expression, respectively, and impaired LC3A/B-II formation (37–39) (*SI Appendix*, Fig. S1*G*). As expected, we observed that depletion of *Atg5* or *Atg7* also resulted in increased MHC-I surface expression (Fig. 1*G*). While PIKfyve inhibition elevated MHC-I surface expression in wild-type cells, it did not further increase surface expression of MHC-I in the *Atg5*-null or *Atg7*-null cancer cells, confirming that PIKfyve inhibition upregulated MHC-I surface expression by impairing autophagy (Fig. 1*G*).

### Genetic or Pharmacologic Inhibition of PIKfyve Upregulates Antigen Presentation and Enhances Cancer Cell Killing by CD8^+^ T Cells.

**Fig. 2.**
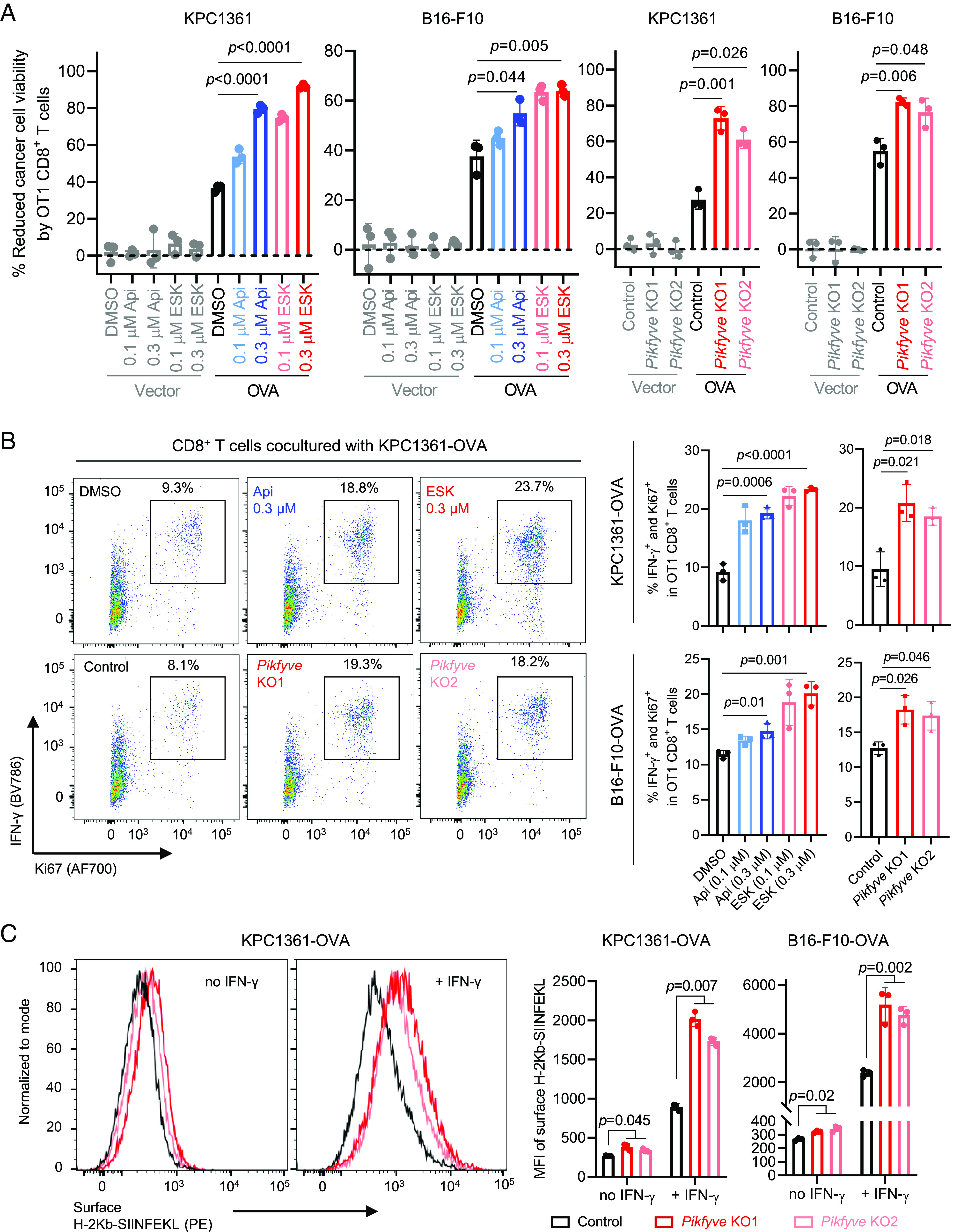
Genetic or pharmacologic inhibition of PIKfyve upregulates antigen presentation and enhances cancer cell killing by CD8^+^ T cells. (*A*) *Left*: Viability of cancer cells with or without OVA overexpression treated with the indicated agent for 24 h, followed by coculture with activated CD8^+^ T cells isolated from OT1 mice for 48 h. *Right*: Viability of control or *Pikfyve*-null cancer cells with or without OVA overexpression, cocultured with activated CD8^+^ T cells isolated from OT1 mice for 48 h. (*B*) Representative images (*Left*) and quantification (*Right*) of flow cytometry measuring the proportion of IFN-γ^+^ and Ki67^+^ cells in the activated CD8^+^ T cells cocultured with the indicated cancer cells in (*A*). (*C*) Representative images (*Left*) and quantification (*Right*) of flow cytometry measuring surface expression of an OVA-peptide (SIINFEKL) bound MHC-I (H-2Kb) in the control or *Pikfyve*-null cancer cells, treated with or without IFN-γ at 10 ng/mL for 24 h. Data in *A*, *B*, and *C* were acquired with three independent biological replicates presented as mean ± SD. Statistics were acquired by two-tailed Student’s *t* test (*A* and *B*) or two-way ANOVA (*C*), with Bonferroni correction. MFI: mean fluorescence intensity. Control: non-targeting single-guide RNA. *Pikfyve* KO1 and *Pikfyve* KO2: independent single-guide RNAs depleting *Pikfyve*.

We next examined whether pharmacologic inhibition or genetic depletion of *Pikfyve* led to enhanced CD8^+^ T cell–mediated cancer cell killing. We generated ovalbumin-expressing (OVA) KPC1361 and B16-F10 cells (*SI Appendix*, Fig. S2 *A* and *B*) and cocultured them with activated CD8^+^ T cells isolated from OT1 mice. We found that the OT1 CD8^+^ T cells had virtually no effect on the control cancer cells expressing the empty vector but killed the OVA-expressing cancer cells (Fig. 2*A*). Importantly, pharmacologic inhibition or genetic depletion of *Pikfyve* significantly enhanced the OVA cancer cell killing mediated by CD8^+^ T cells (Fig. 2*A*). Concordantly, we also observed an increase in IFN-γ expression and Ki-67 proliferating CD8^+^ T cells in co-culture following pharmacologic inhibition or genetic depletion of *Pikfyve* (Fig. 2*B*). We further evaluated the antigen presentation in the OVA cancer cells with flow cytometry, using an antibody detecting an OVA-peptide (SIINFEKL)-bound MHC-I (H-2Kb). As expected, we found that genetic or pharmacologic inhibition of PIKfyve resulted in upregulation of antigen presentation in the cancer cells (Fig. 2*C* and *SI Appendix*, Fig. S2*C*). Collectively, genetic or pharmacologic inhibition of PIKfyve upregulates antigen presentation and enhances cancer cell killing mediated by CD8^+^ T cells.

### Loss of *Pikfyve* Retards Tumor Progression in a CD8^+^ T Cell- and MHC Class I-Dependent Manner.

**Fig. 3.**
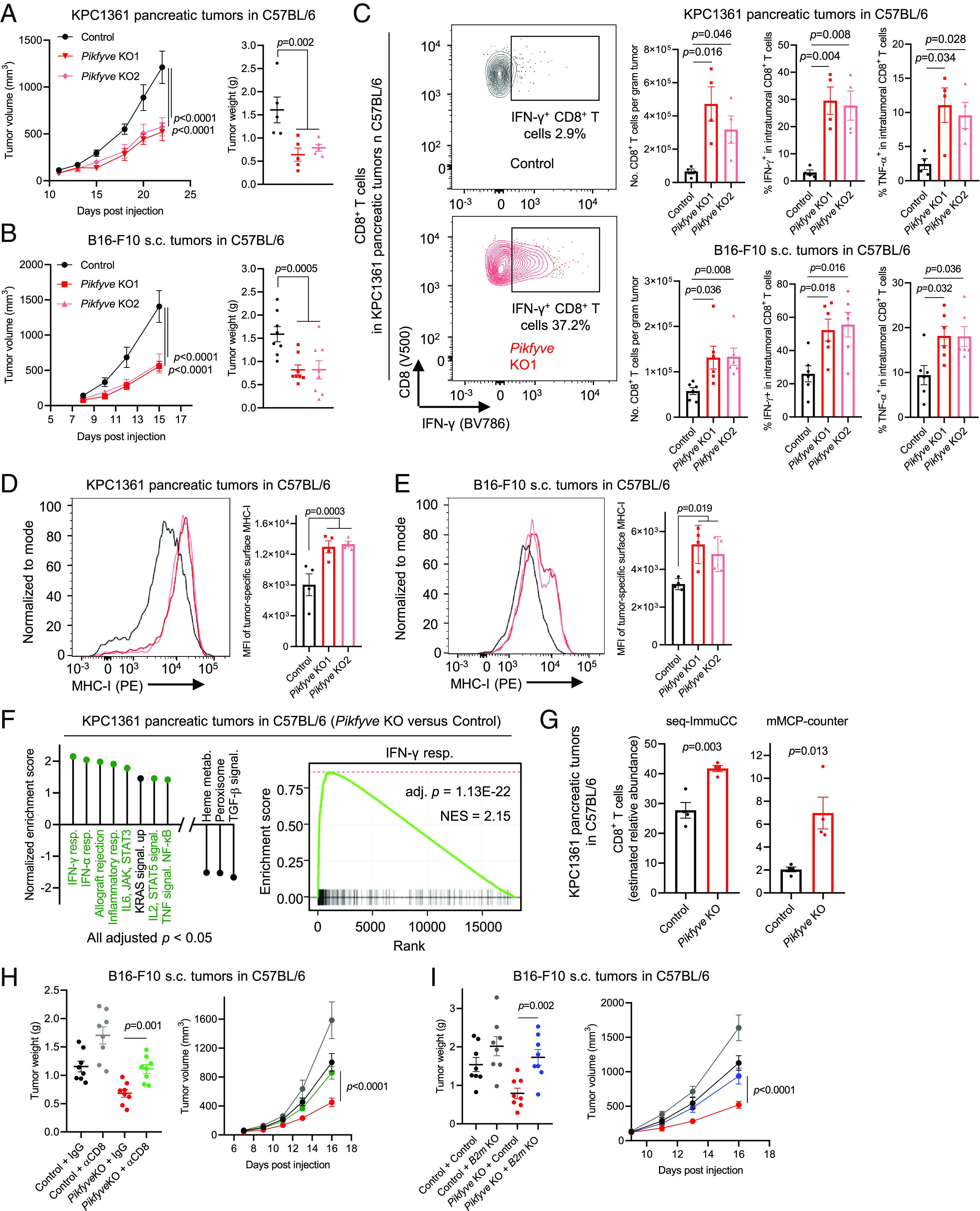
Loss of *Pikfyve* retards tumor growth in a CD8^+^ T cell- and MHC class I-dependent manner. (*A*) Volumes (*Left*) and weights (*Right*) of pancreatic tumors established with orthotopic injection of control or *Pikfyve*-null KPC1361 cells to C57BL/6 mice (*n* = 5 mice, per group). (*B*) Volumes (*Left*) and weights (*Right*) of tumors established with s.c. injection of control or *Pikfyve*-null B16F10 cells to C57BL/6 mice (*n* = 4 mice, per group). Data in A and B are representative of two independent experiments. (*C*) Representative images (*Left*) and quantification (*Right*) of flow cytometry measuring the amount of total CD8^+^ T cells and proportion of activated or proliferative CD8^+^ T cells in the indicated tumors established as in *A* and *B* (KPC1361, *n* = 4 mice per group; B16-F10, *n* = 6 mice per group). (*D* and *E*) Representative images (*Left*) and quantification (*Right*) of flow cytometry measuring surface expression of MHC-I in fluorescent-labelled cancer cells in the indicated tumors established as in *A* and *B* (*n* = 4 mice, per group). (*F*) *Left*: Hallmark pathways enriched by bulk RNA-sequencing of the indicated Pikfyve-KO tumors versus control. Immune-related pathways are highlighted in green. *Right*: Enrichment plot of the top pathway on the left. (*G*) Amount of CD8+ T cells in the indicated tumors estimated by the indicated models, using bulk RNA-sequencing data in (*F*). (*H*) Weights (*Left*) and volumes (*Right*) of tumors established with s.c. injection of control or *Pikfyve*-knockout B16-F10 cells to C57BL/6 mice, with (αCD8) or without (IgG) CD8^+^ T cell depletion (*n* = 4 mice, per group). Data were reproducible with independent *Pikfyve*-knockout single-guide RNA. (*I*) Weights (*Left*) and volumes (*Right*) of tumors established with s.c. injection of control, *B2m* or *Pikfyve* single knockout, or *B2m* and *Pikfyve* double knockout B16-F10 cells to C57BL/6 mice (*n* = 4, per group). Data were reproducible with independent *B2m*-knockout single-guide RNA.All data are presented as mean ± SEM. Statistics were acquired by two-tailed Student’s *t* test in *C*, *G*, and *H* (*Left*), *I* (*Left*), with Bonferroni correction, or two-way ANOVA in *A*, *B*, *D*, *E*, and *H* (*Right*), and *I* (*Right*). Data in (*A*) and (*B*) are representative of two independent experiments. MFI: mean fluorescence intensity. Control: non-targeting single-guide RNA. *Pikfyve* KO1 and *Pikfyve* KO2: independent single-guide RNAs depleting *Pikfyve*.

We next evaluated the effect of *Pikfyve* loss in vivo by injecting the *Pikfyve* knockout cancer cells into their immunocompetent syngeneic hosts (Fig. 3 *A* and *B*). We found that depletion of *Pikfyve* strongly retarded tumor progression in both the pancreatic (KPC1361) and melanoma (B16F10) models, compared to the *Pikfyve*-wild-type control (Fig. 3 *A* and *B*). We also measured the amount of total and activated CD8^+^ T cells in the tumors and observed that knockout of *Pikfyve* increased the amount of intratumoral CD8^+^ T cells and the proportion of activated CD8^+^ T cells (Fig. 3*C* and *SI Appendix*, Fig. S3*A*). Of note, we did not observe any significant change of CD8^+^ T cell activation in the tumor-draining lymph nodes (TdLN; see *SI Appendix*, Fig. S3*B*), suggesting that *Pikfyve*-loss in malignant cells affects quantity and functionality of CD8^+^ T cells in the tumor microenvironment, rather than CD8^+^ T cell priming in TdLN. We further performed flow cytometry to measure the surface expression of MHC-I in cancer cells in vivo using fluorescent-labelled cancer cells (*SI Appendix*, Fig. S3*C*). Consistent with what was observed in vitro, we found that depletion of *Pikfyve* led to upregulation of MHC-I surface expression in tumor cells (Fig. 3 *D* and *E*). Importantly, upregulation of tumor-specific MHC-I expression by *Pikfyve*-loss was also validated using immunofluorescence (*SI Appendix*, Fig. S3*D*). Finally, we performed bulk RNA-sequencing and identified multiple immune-related pathways enriched in the *Pikfyve-*loss tumors when compared to the *Pikfyve-*wild-type, with IFN-γ response being the top pathway (Fig. 3*F*). Estimation of CD8^+^ T cell abundance with different models, seq-ImmuCC (40) or mMCP-counter (41), on the bulk RNA-sequencing data also showed an increase of intratumoral CD8^+^ T cells (Fig. 3*G*), consistent with the flow cytometry data. Collectively, these data suggest that *Pikfyve*-depletion controlled tumor growth in an immune-dependent manner.

The increase of activated CD8^+^ T cells in *Pikfyve*-knockout tumors prompted us to evaluate whether CD8^+^ T cells were essential for the reduction of tumor growth by *Pikfyve*-knockout. We thus depleted CD8^+^ T cells in the syngeneic hosts and evaluated the growth of tumors established with the *Pikfyve*-knockout cancer cells. Successful depletion of CD8^+^ T cells was determined by flow cytometry (*SI Appendix*, Fig. S4*A*). We found that CD8^+^ T cell depletion significantly rescued the progression of tumors derived from *Pikfyve*-knockout cancer cells (Fig. 3*H* and *SI Appendix*, Fig. S4*B*). These data highlight that the tumor control mediated by *Pikfyve*-loss was CD8^+^ T cell dependent. We next sought to determine if the upregulation of MHC-I surface expression by *Pikfyve*-depletion was functional in tumor control. We thus knocked out *B2m*, a molecule crucial for MHC-I assembly (14), in the *Pikfyve*-wild-type or *Pikfyve*-loss cancer cells (*SI Appendix*, Fig. S4*C*). We observed that loss of *B2m* significantly rescued the progression of tumors derived from *Pikfyve*-null cancer cells (Fig. 3*I* and *SI Appendix*, Fig. S4*D*), supporting that the tumor control mediated by *Pikfyve*-loss was also mediated by tumor-specific MHC-I expression. We noticed that neither CD8^+^ T cell depletion nor *B2m* knockout exhibited full rescue of the tumor growth from *Pikfyve*-loss (*SI Appendix*, Fig. S4 *B* and *D*), suggesting that *Pikfyve*-loss may inhibit tumor growth in an immune-independent manner (30). Indeed, cells with *Pikfyve*-depletion were less proliferative than the control in vitro (*SI Appendix*, Fig. S4*E*). Collectively, our data underscored that tumor control by *Pikfyve*-knockout was mediated, at least partially, by CD8^+^ T cells and tumor-specific MHC-I expression.

### PIKfyve Inhibition Enhances Efficacy of ICB, and *PIKFYVE* Expression Predicts Response to ICB and Survival in ICB-Treated Cohorts.

**Fig. 4.**
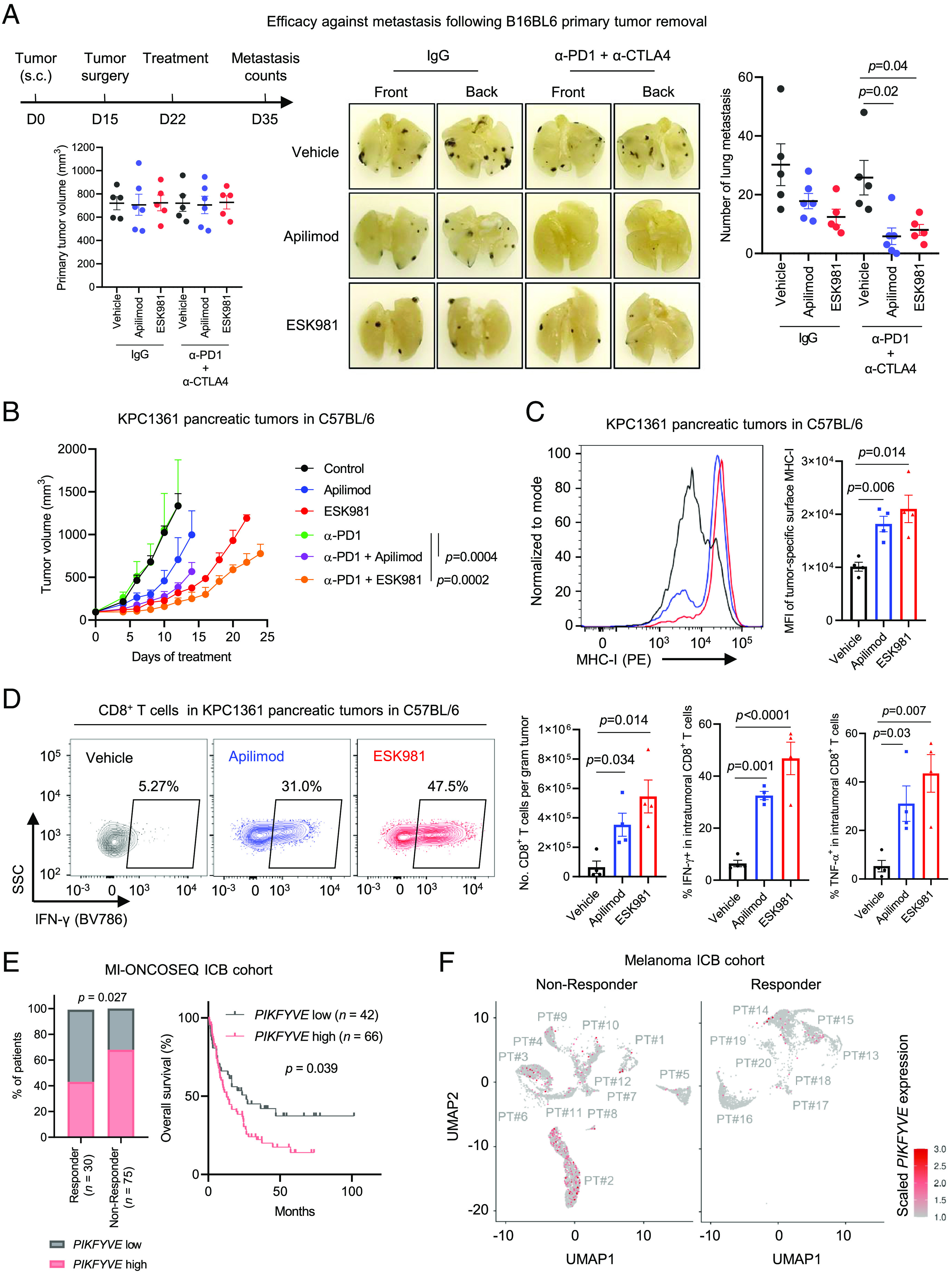
PIKfyve inhibition enhances efficacy of ICB. (*A*) Efficacy of ICB in combination with apilimod or ESK981 against metastasis following B16-BL6 s.c. primary tumor removal. *Top* *Left*: A schematic showing design of the experiment. *Bottom* *Left*: Volumes of primary tumors in each group at randomization when the primary tumors were surgically removed. *Middle*: Representative images of lungs bearing metastasis tumors in the indicated groups, at end point. *Right*: Counts of the lung metastasis in the indicated groups. ESK981 or apilimod were administrated once daily at 30 mg/kg or 60 mg/kg, respectively. Anti-PD1 (α-PD1) and anti-CTLA4 (α-CTLA4) were administrated biweekly at 150 µg/mouse or 100 µg/mouse, respectively. *n* = 5 mice per group, except for the apilimod-treated (*n* = 6 mice, per group). (*B*) Efficacy of α-PD1 in combination with apilimod or ESK981 against pancreatic tumors established with orthotopic injection of KPC1361 cells to C57BL/6 mice. *n* = 4 mice per group, except for the apilimod-treated (*n* = 5 mice, per group). ESK981 or apilimod were administrated as in A. Anti-PD1 (α-PD1) was administrated biweekly at 250 µg/mouse. (*C*) Representative images (*Left*) and quantification (*Right*) of flow cytometry measuring surface expression of MHC-I on GPF-labelled cancer cells in the indicated tumors established as in *B*, 24 h post ESK981 or apilimod administration at 30 or 60 mg/kg, respectively (*n* = 4 mice per group). (*D*) Representative images (*Left*) and quantification (*Right*) of flow cytometry measuring the amount of total CD8^+^ T cells, and proportion of activated or proliferative CD8^+^ T cells in the indicated tumors, established as in *B*, following 2 d of once daily ESK981 or apilimod administration at 30 or 60 mg/kg, respectively (*n* = 4 mice per group). (*E*) *Left*: Proportion of patients with tumors showing high or low pretreatment *PIKFYVE* mRNA levels in complete response (CR) group or not CR group. *Right*: Overall survival of patients with tumors showing high or low pretreatment *PIKFYVE* mRNA levels in a cohort treated with ICB at University of Michigan, Ann Arbor (MI-ONCOSEQ ICB cohort). (*F*) Single-cell RNA-sequencing measuring pre-treatment *PIKFYVE* expression in malignant cells in an ICB-treated melanoma cohort. PT#: Patient number. All data are presented as mean ± SEM, expect for *B* (mean + SEM). Statistics were acquired by two-tailed Student’s *t* test, with Bonferroni correction in (*A*, *C*, and *D*), by Fisher’s exact test in *E* *Left*, and log-rank test in *E* *Right*.

We next sought to determine whether inhibition of PIKfyve improved efficacy of ICB. As metastasis is the major cause of cancer deaths, we first evaluated the efficacy of combining PIKfyve inhibition and ICB against metastasis, using the B16-BL6 melanoma model that has a high inclination to form early lung metastatic dissemination after surgical removal of primary tumors (42). We surgically removed the primary tumors derived from subcutaneous (s.c.) injection of B16-BL6 cells and randomized the mice into different treatment groups based on volumes of the primary tumors (Fig. 4 *A*, *Left*). The mice were treated with ICB (anti-PD-1 plus anti-CTLA-4) or PIKfyve inhibitor alone, or in combination, and killed at end point for quantifying lung metastasis counts (Fig. 4 *A*, *Top* *Left*). As expected, in the control group, significant amounts of lung metastasis were observed at the end point (Fig. 4 *A*, *Middle* and *Right*). The B16-BL6 model was previously reported to be relatively resistant to ICB (43, 44). In line with this, we observed no significant reduction of metastasis in the ICB-treated group (Fig. 4 *A*, *Middle* and *Right*). While the single agent PIKfyve inhibitors (apilimod or ESK981) exhibited reduction of lung metastasis, they also strongly enhanced the efficacy of ICB to inhibit metastasis (Fig. 4 *A*, *Middle* and *Right*). These data highlight the strong efficacy of combining PIKfyve inhibition and ICB against metastasis.

We also found that PIKfyve inhibition improved anti-PD-1 efficacy against syngeneic pancreatic tumors established with orthotopic injection of KPC1361 cells (Fig. 4*B*), which translated into improved overall survival (*SI Appendix*, Fig. S5*A*). Importantly, PIKfyve inhibition by apilimod or ESK981 also led to upregulation of tumor-specific MHC-I surface expression (Fig. 4*C*) and an increase in intratumoral activated CD8+ T cells (Fig. 4*D*). Importantly, PIKfyve inhibitor treatment showed a favorable safety profile as a single agent or in combination with anti-PD-1, as the treated mice showed no perceptible weight loss (*SI Appendix*, Fig. S5*B*).

These data prompted us to determine whether *PIKFYVE* expression in human tumors predicts response to ICB and survival in ICB-treated cohorts. Using pre-treatment RNA-sequencing data from tumors obtained from a stage IV ICB-treated pan-cancer cohort (*n* = 108; Dataset S1) at the University of Michigan (U-M), we found that high pre-treatment *PIKFYVE* expression was significantly associated with poorer response to ICB (Fig. 4*E*). Importantly, high pre-treatment *PIKFYVE* expression also significantly predicted poorer overall survival in this cohort (Fig. 4*E*). Cox regression analysis further revealed that high pre-treatment *PIKFYVE* expression was associated with poorer clinical outcome (*P* = 0.01) independently of age, sex, race, and cancer type (*SI Appendix*, Fig. S5*C*). To validate this observation with public data, we downloaded data from pre-treatment ICB-treated cohorts (*n* = 807) comprised of various cancer types, including bladder (*n* = 73), esophageal adenocarcinoma (*n* = 73), melanoma (*n* = 317), urothelial (*n* = 348), and glioblastoma (*n* = 14). We confirmed that high pre-treatment *PIKFYVE* expression was prognostic of poor overall survival (*SI Appendix*, Fig. S5*D*).

To determine whether *PIKFYVE* expression specifically in malignant cells predicts clinical outcomes of ICB-treated patients, we exploited published single-cell RNA-sequencing datasets representing cancer patients treated with ICB (45–47). We found that pre-treatment tumor specimens from melanoma patients not responsive to ICB displayed higher proportions of malignant cells expressing high *PIKFYVE*, compared to responders (Fig. 4*F*). Furthermore, patients with high pre-treatment *PIKFYVE* expression in malignant cells were less likely to develop expansion of intratumoral T cells after ICB treatment (*SI Appendix*, Fig. S5 *E*, *Left*) or were more likely to show poorer response to ICB treatment (*SI Appendix*, Fig. S5 *E*, *Middle*), highlighting that high pre-treatment *PIKFYVE* expression in malignant cells was significantly associated with unfavourable clinical outcomes in ICB cohorts (*SI Appendix*, Fig. S5 *E*, *Right*).

### PIKfyve Inhibition Enhances Efficacy of ACT and Therapeutic Vaccine Therapy.

**Fig. 5.**
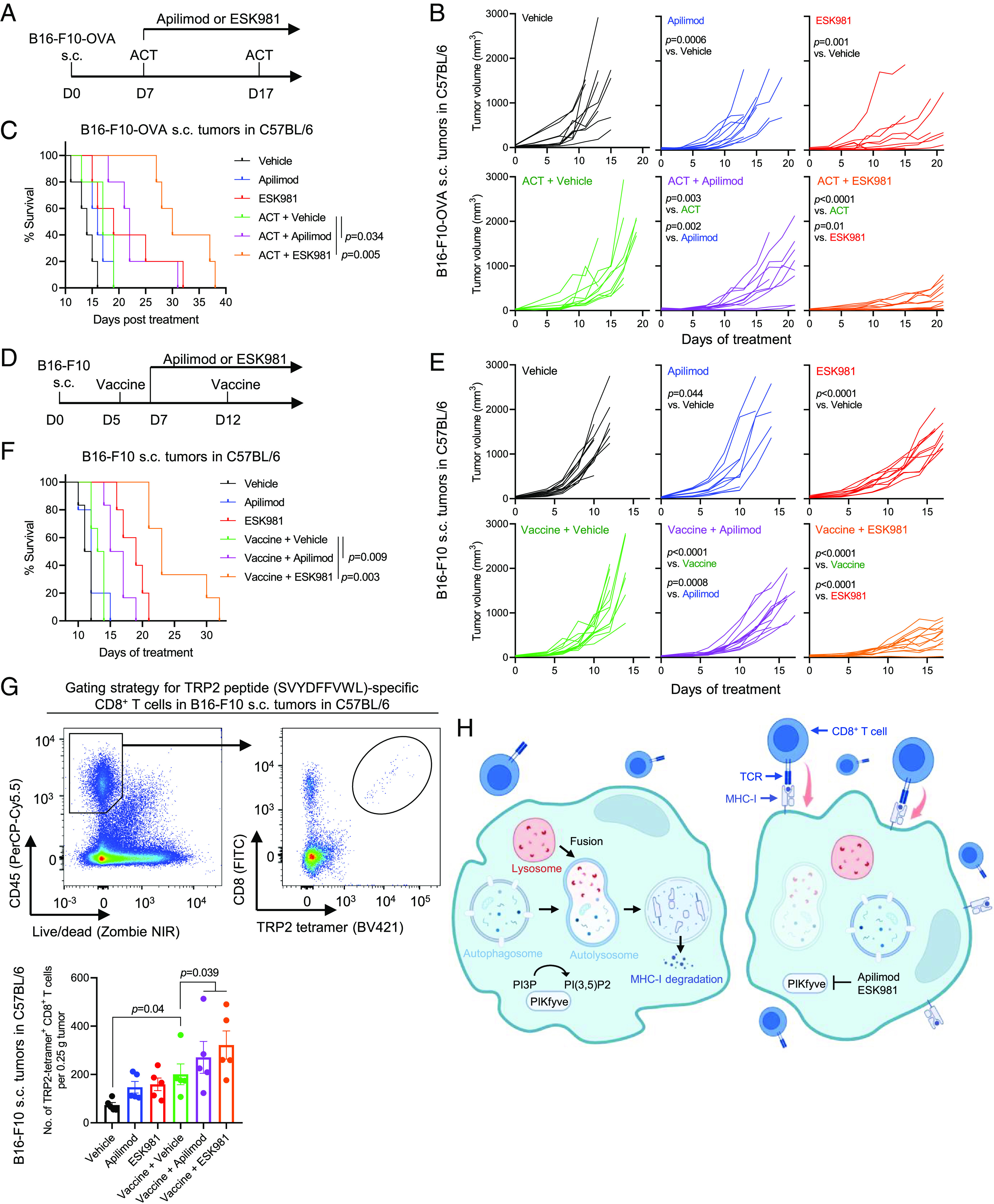
PIKfyve inhibition enhances efficacy of ACT and therapeutic vaccine. (*A*) Schematic of experiment assessing efficacy of ACT in combination with apilimod or ESK981 against s.c. tumors derived from ovalbumin-expressing B16-F10 (B16-F10-OVA). Three and a half million CD8^+^ T cells were isolated from OT1 mice, and cells were then activated in vitro by anti-CD3/CD28 coated beads as the ACT in this experiment. (*B*) Individual growth curves of tumors in mice treated as in (*A*). (*C*) Survival curves of mice in (*B*). (*D*) Schematic of experiment assessing efficacy of therapeutic vaccine in combination with apilimod or ESK981 against s.c. tumors derived from B16-F10. One dose of vaccine was composed of two million irradiated B16-F10 (35 Gy) and 30 µg poly(I:C). (*E*) Individual growth curves of tumors in mice treated as in (*D*). (*F*) Survival curves of mice in (*E*). Statistics were acquired by two-way ANOVA in (*B* and *E*), or log-rank test in (*C* and *F*), with Bonferroni correction. (*G*) *Top*: Gating strategy in flow cytometry identifying TRP2-tetramer^+^ CD8^+^ T cells. *Bottom*: Amount of TRP2-tetramer^+^ CD8^+^ T cells in tumors in mice treated as in (*D*). Tumors were harvested 4 d after the first dose of therapeutic vaccine (*n* = 6 mice per group). (*H*) A schematic showing that PIKfyve inhibition enhances CD8^+^ T cell–mediated anticancer immunity via blocking autophagy-lysosomal MHC-I degradation. Data are presented as mean ± SEM. Statistics were acquired by two-tailed Student’s *t* test and two-way ANOVA with Bonferroni correction.

We next determined whether PIKfyve inhibition could enhance efficacy of other types of immunotherapies by first employing the OVA-expressing cancer cells (*SI Appendix*, Fig. S2 *A* and *B*). To assess whether PIKfyve inhibition enhances efficacy of ACT, we established s.c. tumors derived from OVA-expressing B16-F10 cells and infused CD8^+^ T cells derived from OT1 mice into the tumor-bearing syngeneic hosts (Fig. 5*A*). Consistent with previous reports, the OT1 T cells alone exhibited marginal efficacy against tumors in this model (48) (Fig. 5*B*), but inhibition of PIKfyve with apilimod or ESK981 strikingly enhanced efficacy of the ACT (Fig. 5*B*), significantly extending survival of the animals (Fig. 5*C*).

Recent clinical trials with personalized therapeutic vaccines showed great promise against cancer (6, 49–51). We next interrogated whether PIKfyve inhibition could enhance efficacy of therapeutic vaccines. We, thus, established s.c. tumors with B16-F10 cells and treated the tumor-bearing mice with lethally irradiated B16-F10 cells plus poly(I:C) as an adjuvant, 5 d post tumor inoculation (Fig. 5*D*). Although the therapeutic vaccine alone showed only a marginal effect on tumor control (Fig. 5*E*), inhibition of PIKfyve strongly improved efficacy of the vaccine (Fig. 5*E*), resulting in significant extension of survival (Fig. 5*F*). To examine whether the vaccine induced antigen-specific CD8^+^ T cells, we measured the amount of intratumoral CD8^+^ T cells bearing a T cell receptor specific for an epitope (SVYDFFVWL) of tyrosinase-related protein 2 (TRP2), an enzyme expressed in B16-F10 cells (52, 53). We observed that the vaccine treatment alone increased the amount of intratumoral antigen-specific CD8^+^ T cells, while treatment with PIKfyve inhibitors further increased the number of CD8^+^ T cells (Fig. 5*G*). Collectively, these data demonstrate that PIKfyve inhibition improves efficacy of ACT and vaccine therapy. Importantly, combining ACT or vaccine therapy with PIKfyve inhibitor treatment did not cause weight loss in the animals, indicating no observable toxicity (*SI Appendix*, Fig. S6 *A* and *B*).

## Discussion

CD8^+^ T cells play a crucial role in antitumor immunity and the clinical success of immunotherapies (9–12), and MHC-I expression on tumor cells presenting antigens is crucial for CD8^+^ T cells to recognize the malignant cells (14, 15). These data are reinforced by the observations that genetic loss or inactivating mutations of *B2M* (15, 49, 54, 55) or IFN-γ pathway genes (54–56) confer resistance to immunotherapies. However, many models, including murine and human, in our hands showed surface expression of MHC-I and responsiveness to IFN-γ, whereby treatment of IFN-γ resulted in increase of MHC-I surface expression (Fig. 1*C* and *SI Appendix*, Fig. S1 *A*–*C*), suggesting that the cancer cells did not completely lose *B2M* or IFN-γ pathway genes. This is consistent with the notion that complete loss or homozygous inactivation of *B2M* or IFN-γ pathway genes is not common in cancer (14), highlighting the alternative mechanisms that cancer cells adopt to downregulate MHC-I (13, 14, 16–19).

Autophagy has been recently reported to downregulate MHC-I expression in PDAC models, as well as several non-small-cell lung cancer cell lines (17). PIKfyve plays an essential role in autophagy and lysosomal adaptation processes, whereby inhibiting PIKfyve impairs autophagy via blocking autophagic flux and exhibits antitumor efficacy (30–33). However, whether and how PIKfyve inhibition affects tumor-specific MHC-I surface expression and, thus, sensitivity to CD8^+^ T cell–dependent immunotherapies remained unexplored until this study. Here, we found that genetic depletion or pharmacologic inhibition of PIKfyve upregulated MHC-I surface expression in cancer cells derived from various lineages. To determine whether PIKfyve inhibition upregulated MHC-I surface expression by impairing autophagy, we disrupted autophagy with a genetic approach by depleting *Atg5* or *Atg7*, or with a pharmacologic approach with bafilomycin or chloroquine treatment. We found that, in cells with autophagy disruption, PIKfyve inhibition failed to elevate MHC-I surface expression. These data support the fact that PIKfyve inhibition upregulates surface MHC-I via impairing autophagy. Furthermore, we observed that *Pikfyve* depletion impaired tumor growth in immunocompetent syngeneic mice, accompanied with an increase of intratumoral activated CD8^+^ T cells and tumor cell membrane MHC-I expression. Importantly, CD8^+^ T cells and tumor-specific MHC-I played a crucial role in the tumor control mediated by *Pikfyve*-depletion, as depletion of CD8^+^ T cells by anti-CD8 antibody or disruption of MHC-I by *B2m*-knockout rescued progression of the tumors derived from *Pikfyve*-null cancer cells. Together, our findings suggest that PIKfyve inhibition impairs autophagy, upregulates surface MHC-I, and, thus, controls tumor growth in a CD8^+^ T cell- and MHC-I-dependent manner (Fig. 5*H*).

Cancer immunotherapies successfully extend survival of some patients, but many patients fail to benefit from therapy (5–8). Given the importance of CD8^+^ T cells and tumor-specific MHC-I in the tumor control mediated by *Pikfyve* depletion, we speculated that PIKfyve inhibition could augment immunotherapies that were CD8^+^ T cell- and MHC class I-dependent. These included ICB, certain types of ACT, and vaccine therapy. In the syngeneic models, we found that oral gavage of the phase I-cleared PIKfyve inhibitors, apilimod or ESK981, significantly augmented ICB in both primary tumor and metastasis settings. Flow cytometry revealed that treatment with the PIKfyve inhibitors increased the number of total and activated CD8^+^ T cells in tumors and elevated tumor-specific MHC-I surface expression. ACT using natural tumor-reactive T cells or T cells with engineered T cell receptor is also MHC class I-dependent and has shown effectiveness against different cancer types in the clinic (3). To address whether PIKfyve inhibition augments ACT, we established tumors derived from OVA-expressing cancer cells and infused OT1 CD8^+^ T cells to the tumor-bearing syngeneic hosts. PIKfyve inhibition strongly improved the efficacy of ACT in this model, translating to significant survival extension of the animals. Recent clinical trials show that personalized vaccine therapies hold great promise against cancer (6, 49–51). We thus also tested the combination of PIKfyve inhibition and vaccine therapy and found that inhibition of PIKfyve significantly improved the efficacy of a vaccine composed of lethally irradiated cancer cells and poly(I:C). Survival of the tumor-bearing mice was also significantly improved by PIKfyve inhibition in the vaccinated groups.

Collectively, our data suggest that treatment with a PIKfyve inhibitor, apilimod or ESK981, enhances efficacy of immunotherapies that are CD8^+^ T cell and MHC class I dependent. Further clinical investigations combining PIKfyve inhibition with immunotherapy, ICB, ACT, or vaccines, are warranted. Importantly, apilimod and ESK981 exhibit favorable safety profiles in our preclinical models, both as single agents and in combination with the immunotherapies tested (*SI Appendix*, Figs. S5*B* and S6 *A* and *B*). We did note that the efficacy of apilimod was weaker than ESK981 in many of our models, even though the dose of apilimod was higher than ESK981. This could be, at least partially, explained by the reported instability of apilimod (57). This highlights the need for developing apilimod derivatives with improved stability. In this study, we also observed that high pre-treatment *PIKFYVE* expression is strongly predictive of poorer response to ICB treatment, and significantly prognostic of poorer overall survival in ICB-treated cohorts. Whether *PIKFYVE* expression serves as a biomarker for immune responses triggered by PIKfyve inhibition also merits further investigation and validation in additional cohorts.

A previous study from our team demonstrated that ESK981 improves efficacy of anti-PD1 therapy in prostate cancer and in one breast cancer model, 4T1, via recruiting intratumoral CD8^+^ T cells (30). Our current study advances the understanding of how PIKfyve mediates immune evasion in cancer, whereby PIKfyve downregulates surface MHC-I, a complex which is crucial for CD8+ T cell recognition and CD8+ T cell–dependent immunotherapies. Therefore, targeting PIKfyve may not just augment ICB in prostate cancer (30), but also improve response to other CD8+ T cell–dependent immunotherapies, such as adoptive cell therapies and therapeutic vaccines, across various cancer types. Of note, a major model tested in this study, KPC1361, is a model of PDAC, a cancer type that is mostly unresponsive to ICB (58). Our data show that KPC1361 pancreatic tumors are resistant to anti-PD1 monotherapy but are sensitized to anti-PD1 by apilimod or ESK981 treatment (Fig. 4*B*). These findings emphasize the potential of inhibiting PIKfyve to sensitize PDAC to immunotherapies.

## Methods

### Cell Lines.

The KPC1361 cell line was generated from a pancreatic tumor of a genetically engineered mouse model (*LSL-Kras^G12D/+^; LSL-Trp53^R172H/+^; Pdx1-Cre*). Briefly, the tumor was cut into small pieces with surgical scissors and digested with collagenase D (Roche; catalog #: COLLD-RO) at 0.5 mg/mL and DNase I (Roche; catalog #: 10104159001) at 0.25 mg/mL, at 37 °C for 45 min. The suspension was then filtered with a 70-µm cell strainer, and tumor cells were enriched and maintained in DMEM, high glucose (Gibco; catalog #: 10566016) supplemented with 10% (v/v) fetal bovine serum (Gibco; catalog #: 16140071) and 50 U/mL penicillin-streptomycin (Gibco; catalog #: 15140-122). Cells used in the study were from passage 8-30. B16-F10, 4T1, MyC-CaP, MIA PaCa2, and LNCaP cell lines were acquired from American Type Culture Collection (ATCC, Manassas, Virginia) while B16-BL6 were acquired from Riken (Japan). All cell lines were authenticated regularly with sequencing by Labcorp Cell Line Testing division (Burlington, North Carolina). All cell lines were maintained at 37 °C in 5% carbon dioxide and 95% atmospheric air. A *mycoplasma* test was performed every 2 wk to ensure that all cells used in the study were *mycoplasma*-free.

### Prostate Patient-Derived Xenograft (PDX) Model.

The PDX model was acquired as described previously (59). Briefly, the model was acquired from a man diagnosed with castration-resistant prostate cancer undertaking cystoprostatectomy. Mixed prostatic adenocarcinoma and neuroendocrine carcinoma was identified by histopathology on the cystoprostatectomy specimen. The tumor was cut into fragments with a 2-mm^3^ size, coated with 100% Matrigel, and implanted to both flanks in a male severe combined immunodeficiency (SCID) mouse. Tumors formed in mice were expanded and maintained in male SCID mice. For apilimod or ESK981 treatment, the tumor-bearing mice were randomized when the tumors approached 100 to 200 mm^3^ in size. Apilimod or ESK981 was administrated as described in *SI Appendix*, *Supplementary Methods*.

### Immunocytochemistry.

Cancer cells were seeded on cover glasses coated with 0.01% poly-L-Lysine (Sigma-Aldrich; catalog #: A-005-C). The cells were then treated with DMSO, apilimod, or ESK981, and stimulated with 10 ng/mL mouse recombinant IFN-γ (R&D Systems; catalog #: 485-MI) for 24 h, followed by fixation with 2% paraformaldehyde in PBS and one washing with PBS. The cells were then permeabilized with 0.25% Triton X-100 in PBS, washed with PBS three times, and blocked with 5% goat serum in PBS for 1 h at room temperature. After blocking, the cells were incubated with the MHC-I antibody (ER-HR52; Novus Biologicals; catalog #: NB100-64952) at 4 °C overnight, followed by PBS wash for three times and incubation of secondary antibody, Alexa Fluor 488 goat anti-rat IgG antibody (Jackson Immunoresearch; # 112-545-167) at room temperature for 1 h. After further washing with PBS three times, the cells were stained with DAPI and mounted on slides for imaging.

### RT-qPCR.

Cells were lysed with QIAzol lysis reagent, and RNAs were extracted with the RNeasy Mini kit (Qiagen), according to the instructions from the manufacturer. The RNAs were next converted into cDNA using the Maxima First Strand cDNA Synthesis kit (Thermo Fisher Scientific; catalog #: K1671), according to the user manual. Quantitative PCR was next conducted with the Fast SYBR Green Master Mix (Thermo Fisher Scientific; catalog #: 4385612) on QuantStudio 5 or 7 Pro system (Thermo Fisher Scientific) in a 386-well plate format. House-keeping gene *ACTB* served as a control for normalization. Relative abundance of the transcripts was examined using 2^−ΔΔC^T. Sequences of the primers used in this study are listed in *SI Appendix,* Table S1.

### Human Studies.

Acquisition and utilization of all clinical data in this study were approved by the U-M Institutional Review Board. Patients were recruited via the U-M Hospital, Ann Arbor, MI, USA, to receive ICB therapy. Patients enrolled in the MI-ONCOSEQ sequencing program (60–63) at the Michigan Center for Translational Pathology (MCTP) and that had sequencing data from pre-treatment tumors were used for prediction of treatment response and survival. Survival times were calculated from the beginning of therapy. RECIST1.140 criteria were used to establish treatment response. Patients with pseudoprogression [imRECIST criteria (64)] were excluded. Sequencing was conducted with approved protocols in the MCTP Clinical Laboratory Improvement Amendments-compliant sequencing laboratory as described previously (61, 62, 65). Briefly, total RNA was purified with the AllPrep DNA/RNA/miRNA kit (Qiagen) and sequenced with the exome-capture transcriptome system in the paired-end method on a HiSeq 2000 or HiSeq 2500 (Illumina). Quality control, alignment, and expression quantification was achieved with CRISP, the standard clinical RNA-Seq pipeline (66). Data were then analysed with the R package *edgeR* (67).

### Analysis of Public Data.

Public data from immunotherapy-treated cohorts with *PIKFYVE* mRNA expression were downloaded from the Kaplan Meier plotter (68) (https://kmplot.com/analysis/index.php?p=service). Data from pre-treatment samples were used for the prediction of survival. Best cutoff was selected in the dichotomized analysis.

### Statistical Analysis.

All data points were acquired with distinct samples rather than acquiring with repeated assessments. Data were analyzed and plotted with Prism version 8 (GraphPad Software; San Diego, CA). Data were presented as means ± SD or ± SEM, as stated in the figure legends. Statistical significance was determined with a *P*-value less than 0.05, unless stated otherwise.

## Supplementary Material

Appendix 01 (PDF)

Dataset S01 (XLSX)

## Data Availability

RNA-sequencing data have been deposited in the Gene Expression Omnibus, accession number GSE235945 (69). All other data are available in the manuscript and/or supporting information.
