# Supplementary material for: Targeting the lipid kinase PIKfyve upregulates surface expression of MHC class I to augment cancer immunotherapy
Source: Proc Natl Acad Sci U S A. 2023 Nov 27;120(49):e2314416120. doi: 10.1073/pnas.2314416120 (PMC10710078; doi:10.1073/pnas.2314416120)
Supplement: Supplementary file 1 — Appendix 01 (PDF) [file pnas.2314416120.sapp.pdf]

PNAS

Supporting Information for

Targeting the lipid kinase PIKfyve upregulates surface expression of MHC class I to augment cancer immunotherapy

Yi Bao[1,2], Yuanyuan Qiao[1,2,3], Jae Eun Choi[1,2], Yuping Zhang[1,2], Rahul Mannan[1,2], Caleb Cheng[1,2], Tongchen He[1,2], Yang Zheng[1,2], Jiali Yu[4,5], Mahnoor Gondal[1,2,6], Gabriel Cruz[1], Sara Grove[4,5], Xuhong Cao[1,2], Fengyun Su[1,2], Rui Wang[1,2], Yu Chang[1,2], Ilona Kryczek[4,5], Marcin Cieslik[1,2,6], Michael D. Green[5,7,8], Weiping Zou[1,2,3,4,5], and Arul M. Chinnaiyan[1,2,3,9,10]

[1] Michigan Center for Translational Pathology, University of Michigan, Ann Arbor, MI, 48109, USA
[2] Department of Pathology, University of Michigan, Ann Arbor, MI, 48109, USA
[3] Rogel Cancer Center, University of Michigan, Ann Arbor, MI, 48109, USA
[4] Department of Surgery, University of Michigan, Ann Arbor, MI, 48109, USA
[5] Center of Excellence for Cancer Immunology and Immunotherapy, University of Michigan, Ann Arbor, MI, 48109, USA
[6] Department of Computational Medicine & Bioinformatics, University of Michigan, Ann Arbor, MI, 48109, USA
[7] Department of Radiation Oncology, University of Michigan, Ann Arbor, MI, 48109, USA
[8] Veterans Affairs Ann Arbor Healthcare System, Ann Arbor, MI, 48109, USA
[9] Howard Hughes Medical Institute, University of Michigan, Ann Arbor, MI, 48109, USA
[10] Department of Urology, University of Michigan, Ann Arbor, MI, 48109, USA

Correspondence to:
Arul M. Chinnaiyan, M.D., Ph.D.
arul@med.umich.edu

This PDF file includes:

Supplementary Methods
Figures S1 to S6
Tables S1 to S2
References

Other Supporting Information for this manuscript include the following:

Data S1 (Excel file)

Supplementary Methods

Stable cell lines with gene knockout or overexpression

Single guide RNAs (sgRNAs) designed to target constitutive exons nearest to the start codon of the target genes were subjected to off-target prediction with Off-Spotter (https://cm.jefferson.edu/Off-Spotter/), and sgRNAs with the lowest off-target potential were used. The list of sgRNAs used in this study can be found in **Table S2**. The sgRNAs were then constructed into lentiCRISPR v2 (Addgene; #52961) with Golden Gate reaction. Briefly, the annealed oligos were mixed with the backbone plasmid in a reaction containing BsmBI and T7 ligase. Following 15 cycles of digestion and ligation, the product from the reaction was used to transform Stbl3 competent cells. Successful insertion of the oligos was confirmed with Sanger sequencing by Eurofins Genomics (Louisville, Kentucky). To generate cells with the gene knockout, the vector containing the sgRNA was transiently transfected into target cells with Lipofectamine 3000 (Thermo Fisher Scientific; catalog #: L3000001) according to the manufacturer's instructions. After puromycin selection, single cells were seeded to 96 well dishes. Colonies from the single cells were then expanded to generate the sublines with the gene knockout. Depletion of the target gene was confirmed by western blot and Sanger sequencing. To generate the ovalbumin-expressing (OVA) cells, cytoplasmic ovalbumin was amplified from pBabe_Hyg_cOVA_T2A_mStrawberry (Addgene; #161737) (1), with PrimeSTAR® Max DNA Polymerase (Takara Bio; catalog #: R045A) and constructed into pLenti CMVie-IRES-BlastR (Addgene; #119863). The constructed vector was next transfected into HEK293T together with pRSV-REV (Addgene; catalog #: 12253), pMDLg/pRRE (Addgene; catalog #: 12251), and pMD2.G (Addgene; catalog #: 12259). Medium containing the virus was collected 72-hours post transfection and filtered with 0.22 µm filter for cell debris removal. Target cells were then transduced with the virus in combination with 4 µg/ml of polybrene (Sigma-Aldrich; catalog #: H9268). One day post viral transduction, the cells were selected with blasticidin S (Thermo Fisher Scientific; catalog #: A1113903) at 4 µg/ml for KPC1361 and 10 µg/ml for B16-F10. The selected cells were used for further experiments.

GFP-labeled cancer cells were generated by transducing virus Lenti-GFP containing CMV-driven GFP to the target cells. The virus was acquired from the Biomedical Research Core Facilities at the University of Michigan (U-M). Two days after the viral transduction, the GFP-positive cells were sorted with a cell

sorter (SONY SH800S). All stable cell lines were tested every two weeks for mycoplasma contamination to ensure that all cells used in the experiments were mycoplasma-free.

Animal studies

All experimental protocols were conducted after approval by the U-M Institutional Animal Care & Use Committee. As previous investigations did not show a difference between sexes in ESK981 efficacy, female C57BL/6 mice aged 6-8-week-old were used in the study. KPC1361 cells were resuspended in PBS, 1:1 (v/v) mixed with Matrigel (BD Biosciences; catalog #: 354248), and orthotopically injected to the pancreases at 250,000 cells in a total volume of 50 µl per injection. B16-F10 cells were resuspended in PBS and injected subcutaneously to both flanks at 400,000 cells per injection. For ACT study, B16-F10-OVA cells were resuspended in PBS and injected subcutaneously to both flanks at 300,000 cells per injection. For lung metastasis experiment, B16-BL6 cells were resuspended in PBS, 1:1 (v/v) mixed with Matrigel and injected subcutaneously to both flanks at 400,000 cells per injection. Fifteen days after tumor cell inoculation, the primary tumors were removed surgically under anesthesia. Thirty-five days after removal of the primary tumors, lung tissues were extracted and fixed, and lung metastasis was quantified. C57BL/6 mice were acquired from The Jackson Laboratory. Measurement of tumor volume began 5-9 days post tumor cell implantation and was performed 2-3 times per week using calipers. Volume measurements of pancreatic tumors were also achieved with calipers, as reported (2). In brief, mice were subjected to anesthesia with 2-3% isoflurane, and the abdominal wall then became soft and loose. The mouse was next held with the left hand allowing for the palpation and assessment of the firm pancreatic tumor mass with the right hand. Mice with KPC1361 pancreatic tumors did not develop ascites in our hands. Volume (mm^3) of tumor was calculated using $(W^2 \times L)/2$, where W stood for minor tumor axis and L for the major. All mice were maintained in a pathogen-free condition, with a 12-hour light/ dark cycle.

***In vivo* treatments**

For ICB treatment in KPC1361 pancreatic tumor-bearing mice, randomization was performed when tumors reached approximately 100 mm^3. For ACT and vaccine experiments, the mice were randomized when tumors reached approximately 35 mm^3. Anti-mouse PD-1 (clone RMP1-14) and its isotype control were purchased from BioXcell, administered intraperitoneally at 250 µg per mouse, biweekly. Apilimod

(MedChemExpress; catalog #: HY-14644) and ESK981, acquired from Esanik Therapeutics, were suspended in Suspending Vehicle Ora-Plus (Perrigo) and administered via oral gavage at 60 and 30 mg/kg, respectively. For ACT, $CD8^+$ T cells were isolated from the spleens and lymph nodes of OT1 mice (The Jackson Laboratory; # 003831) with Mouse $CD8^+$ T Cell Isolation Kit (Stemcell; catalog #: 19853). The cells were then activated and expanded with the mouse T Cell Activation/Expansion kit (Miltenyibiotec; # 130-093-627) in T cell-medium containing RPMI 1640 (Gibco; catalog #: 11875093) supplemented with 10 mM HEPES, 10% (v/v) fetal bovine serum (Gibco; catalog #: 16140071), 50 U/ml penicillin-streptomycin (Gibco; catalog #: 15140-122), 27.5 µM beta-mercaptoethanol (Sigma; catalog #: M3148-100ML), and 10 ng/ml mouse recombinant IL-2 (STEMCELL; catalog #: 78081.1), for four days, with medium and activation beads refreshed on day two. The expanded T cells were collected and washed with PBS. The T cells in PBS were next intravenously injected to the animals, on day seven and day 17 post tumor cell inoculation. For vaccine therapy, B16-F10 cells were resuspended in HBSS (HyClone) and irradiated at 35 Gy, and subcutaneously injected with poly(I:C) at two-million irradiated cancer cells plus 30 µg poly(I:C) (Invitrogen; catalog #: vac-pic) per mouse, on day five and day 12 post tumor cell inoculation. For $CD8^+$ T cells depletion, anti-mouse CD8α (clone 2.43), and its isotype controls were purchased from BioXcell, and administrated intraperitoneally on days 2, 5, 8, 11, and 14 post tumor cell inoculation, at 400 µg per mouse as a loading dose and 100 µg per mouse subsequently. Efficacy of CD8+ T cell depletion was assessed by flow cytometry with blood isolated from the treated mice. No blinding was used in the study.

Coculture of cancer cells and $CD8^+$ T cells

$CD8^+$ T cells were isolated from OT1 mice and activated for four days *in vitro* as described above. The OVA-expressing cancer cells were established as described above. Cancer cells with *Pikfyve* knockout were directly cocultured with the activated $CD8^+$ T cells, while the apilimod- or ESK981-treated cells were treated with the agent at the indicated concentrations for 24 hours prior to the coculture. To establish the coculture, the cancer cells were seeded into 96 well dishes at 5,000 cells per well, and then 5,000 activated $CD8^+$ T cells were added into the well. Two days after the coculture, the $CD8^+$ T cells were collected for flow cytometry analysis. After removal of the $CD8^+$ T cells, the viable cancer cells were measured by MTT (3-(4,5-Dimethylthiazol-2-yl)-2,5-Diphenyltetrazolium Bromide; Thermo Fisher Scientific; catalog # M6494), according to the instructions from the manufacturer. For models

derived from B16-F10, the coculture was performed in the above mentioned T cell-medium. For models derived from KPC1361, the coculture was performed in 45% DMEM, high glucose (Gibco; catalog #: 10566016), 45% RPMI 1640 (Gibco; catalog #: 11875093) supplemented with 10% (v/v) fetal bovine serum (Gibco; catalog #: 16140071), 10 mM HEPES, 50 U/ml penicillin-streptomycin (Gibco; catalog #: 15140-122), 27.5 μM beta-mercaptoethanol (Sigma; catalog #: M3148-100ML), and 10 ng/ml mouse recombinant IL-2 (STEMCELL; catalog #: 78081.1).

Flow cytometry analysis

Cancer cells or the OVA-expressing cancer cells treated *in vitro* were harvested from dishes, resuspended in buffer MACS (PBS containing 2% FBS and 2 mM EDTA), and stained with Zombie NIR™ Fixable Viability kit (BioLegend: # 423106) and fluorophore-conjugated antibody against MHC-I or PE-conjugated antibody against SIINFEKL bound H-2Kb (BioLegend; catalog # 141604), respectively. The cells were next washed twice with 1 ml MACS and fixed with 2% paraformaldehyde in PBS for 15 minutes at room temperature. The cells were then subjected to the flow cytometer (SONY SH800S) for measuring surface expression of MHC-I or SIINFEKL bound H-2Kb, respectively. Antibodies used in this study include anti-H-2Kb (BD Biosciences; catalog # 553570) and anti-H-2Db (28-14-8; Thermo Fisher Scientific; catalog # 12-5999-82) for KPC1361 and B16-F10, anti-H-2Kd (BD Biosciences; catalog # 562004) and anti-H-2Dd (BD Biosciences; catalog # 553580) for 4T1, anti-H-2Kq (BD Biosciences; catalog # 742296) and anti-H-2Dq/H-2Lq (BD Biosciences; catalog # 744853) for MyC-CaP, and anti-HLA-A,B,C (clone w6/32; BioLegend; catalog # 311406) for human cancer cells. For measuring the proliferation and functionality of $CD8^+$ T cells in the coculture of cancer cells and OT1 $CD8^+$ T cells, the T cells were collected from the coculture and stimulated in the above mentioned T cell-medium supplemented with a stimulation cocktail containing 200 ng/ml PMA (Phorbol 12-Myristate 13-Acetate; Sigma; catalog # P1585), 1 μg/ml ionomycin (Sigma; catalog # IO634), 1X brefeldin A (Thermo Fisher Scientific; catalog # 00-4506-51) and 1X monensin (Thermo Fisher Scientific; catalog # 00-4505-51) at 37 ℃ for four hours. The cells were then collected, resuspended in MACS, and stained with Zombie Green™ Fixable Viability kit (BioLegend; catalog #423111) and anti-CD8 antibody (BD Biosciences; catalog # 560776). After washing with MACS, the cells were next fixed/permeabilized, washed with the Foxp3/Transcription Factor Staining Buffer Set (Thermo Fisher Scientific; catalog # 00-5523-00), and stained with anti-Ki67 (Thermo Fisher Scientific; catalog # 56-

5698-82) and anti-IFN-γ (BD Biosciences; catalog # 563773). After further washing with MACS, the cells were subjected to flow cytometry assessment on the BD LSRFortessa™ Cell Analyzer.

For profiling T cells in tumors or tumor-draining lymph nodes, the tissues were cut into small pieces with a blade and digested with collagenase D (Roche; catalog #: COLLD-RO) plus DNase I (Roche; catalog #: 10104159001) at 0.5 and 0.25 mg/ml, respectively, at 37 ℃ for 40 minutes with agitation. Following filtering with 70 μm cell strainers, the suspensions were carefully layered into centrifuge tubes containing density gradient medium (Lymphoprep; STEMCELL; catalog # 07851). After centrifuge, the mononuclear cell layer at the interface was collected and washed once with MACS. The cells were then stimulated with the above-mentioned cocktail containing PMA (Phorbol 12-Myristate 13-Acetate), ionomycin, brefeldin A, and monensin in the T cell-medium at 37°C for four hours. The cells were next washed once with MACS and blocked with anti-mouse CD16/32 (Biolegend; catalog #: 156604) at room temperature for 5 minutes and stained with anti-CD45 (BD Biosciences; catalog # 550994), anti-CD3 (BD Biosciences; catalog # 555274), anti-CD90 (BioLegend; catalog # 140327), and anti-CD8 (BD Biosciences; catalog # 560776), at room temperature for 15 minutes. After staining, the cells were washed once with MACS, and fixed/permeabilized and washed with the Foxp3/Transcription Factor Staining Buffer Set (Thermo Fisher Scientific; catalog # 00-5523-00). The cells next were stained for anti-Ki67 (Thermo Fisher Scientific; catalog # 56-5698-82), anti-TNF-a (BioLegend; catalog # 506324), and anti-IFN-γ (BD Biosciences; catalog # 563773) at room temperature for 15 minutes and washed twice with MACS. Absolute Counting Beads (Thermo Fisher Scientific; catalog #: C36950) were then added to the samples for quantification of the target cells according to the instructions from the manufacturer, and the samples were next subjected to flow cytometry assessment on the BD LSRFortessa™ Cell Analyzer. For TRP2 tetramer staining, the tumor tissues were cut, digested, and filtered as described above. The suspensions were then stained with the Zombie NIR™ Fixable Viability kit (BioLegend: # 423106) and Tetramer/BV421 H-2Kb TRP2 (SVYDFFVWL; MBL International; # TB-5004-4) at room temperature for 10 minutes and blocked with anti-mouse CD16/32 (Biolegend; catalog #: 156604) at room temperature for 5 minutes. After blocking, the suspensions were stained with anti-CD45 (BD Biosciences; catalog # 550994) and anti-CD8 (BD Biosciences; catalog # 560776) for 15 minutes at room temperature. Red blood cells were then lysed with the RBC Lysis Buffer (BioLegend; catalog #: 420301) at room temperature for four minutes, and the cells were then washed twice with

MACS and fixed in 0.5% paraformaldehyde in PBS at 4°C for one hour. Absolute Counting Beads (Thermo Fisher Scientific; catalog # C36950) were next added to the samples for quantification of the target cells according to the instructions from the manufacturer, and then the samples were subjected to flow cytometry assessment on the BD LSRFortessa™ Cell Analyzer. For measuring MHC-I surface expression in tumor cells, tumors derived from GFP-labeled cancer cells were cut, digested, and filtered as described above. The suspensions were then stained with the Zombie NIR™ Fixable Viability kit (BioLegend: catalog #: 423106) and anti-H-2Kb (BD Biosciences; catalog # 553570) plus anti-H-2Db (28-14-8; Thermo Fisher Scientific; catalog #: 12-5999-82) as described above. The suspensions were next washed and fixed as described above and subjected to BD LSRFortessa™ Cell Analyzer for MHC-I measurement. For measuring $CD8^+$ T cells post anti-CD8α antibody treatment, 100 μl of blood was collected from the tail into a tube containing 40 μl 0.5 M EDTA solution (pH 8.0). The suspension was blocked with anti-mouse CD16/32 (Biolegend; cat. no. 156604) as described above and stained with anti-CD45 (BD Biosciences; catalog #: 550994), anti-CD3 (BD Biosciences; catalog #: 555274), anti-CD90 (BioLegend; catalog #: 140327), anti-CD8 (BD Biosciences; catalog #: 560776), and anti-CD4 (BD Biosciences; catalog #: 553051), for 15 minutes at room temperature. Red blood cells were then lysed with the RBC Lysis Buffer (BioLegend; # 420301), followed by washing with MACS twice. The samples were then fixed with 2% paraformaldehyde in PBS for 15 minutes, followed by assessment on the BD LSRFortessa™ Cell Analyzer. All flow cytometry data were analysed with FlowJo V10.8.1.

Immunofluorescence

Dissected tumor tissues were frozen in TissueTek OCT Compound (Sakura Finetek) by liquid nitrogen. The frozen sample blocks were cut into 5 μm sections and then fixed at room temperature with 2% paraformaldehyde for 15 mins. Permeabilization was then performed on the sections with 0.25% Triton X-100 for 15 mins. After washing with PBS three times, the sections were next blocked with 5% goat serum and incubated with the MHC-I antibody (ER-HR52; Novus Biologicals; catalog #: NB100-64952) at 4°C overnight, followed by three PBS washes and incubation of secondary antibody, Alexa Fluor 488 goat anti-rat IgG antibody (Jackson Immunoresearch; # 112-545-167) for one hour at room temperature. After washing with PBS three times, the sections were stained with DAPI and mounted on slides for imaging. Quantification for MHC-I levels was performed using the Fiji Is Just ImageJ downloadable online (https://imagej.net/software/fiji/downloads).

Immunohistochemistry

Following paraffin-embedding, xenograft tissues were sectioned and deparaffinized, followed by rehydration. Antigen retrieval was then performed in sodium citrate buffer (10 mM sodium citrate, 0.05% Tween 20, pH 6.0), followed by treatment of 3% H_2O_2 and blocking in PBS containing 5% goat serum. The sections were then incubated with the primary antibody, anti-HLA class 1 ABC (EMR8-5; Abcam; catalog #: ab70328) overnight at 4 ℃. The sections were then washed with PBST (0.1% Tween 20 in PBS), followed by incubation with the secondary antibody, Goat Anti-Rabbit IgG (H + L)-HRP Conjugate (Bio-Rad; #1706515). The sections were further washed with PBST, counterstained with hematoxylin, and imaged using a microscope. Quantification for MHC-I levels was performed after deconvoluting the brown layer from the image using the Fiji Is Just ImageJ downloadable online (https://imagej.net/software/fiji/downloads).

Immunoblot

Cells were lysed in RIPA buffer (Thermo Fisher Scientific; catalog #: 89901) supplemented with a protease inhibitor cocktail (Cell Signaling Technology; catalog #: 5871). The lysates were then sonicated for a total of 30 seconds. Cell debris was then removed by centrifugation, and the protein concentration was measured with the Pierce™ BCA Protein Assay Kit (Thermo Fisher Scientific; catalog #: 23227) according to the manufacturer's instructions. The samples were next loaded and separated by SDS-PAGE and transferred to PVDF membrane (Merck; catalog #: IPVH00010). After blocking with 5% (w/v) BSA or milk, the membrane was incubated with primary antibody at 4°C overnight and washed three times with TBST (0.1% Tween 20 in TBS). The membrane was then blotted with horseradish peroxidase (HRP)-linked secondary antibody and washed with TBST for another three times. Chemiluminescent substrate (Thermo Fisher Scientific; catalog #: 34096) was then applied to the membrane for visualization on the ChemiDocTM XRS + Imaging System (Bio-Rad). Antibodies used in this study include anti-PIKfyve (R&D Systems; catalog #: AF7885), anti-LC3A/B (Cell Signaling Technology; catalog #: 12741S), anti-GAPDH (Cell Signaling Technology; catalog #: 3683S), anti-Vinculin (Cell Signaling Technology; catalog #: 4650S), anti-H3 (Cell Signaling Technology; catalog #: 3638S), anti-MHC-I (Abcam; catalog #: ab70328), anti-ATG5 (Cell Signaling Technology; catalog #: 12994S), anti-ATG7 (Cell Signaling Technology; catalog #: 8558S), anti-ovalbumin (Thermo Fisher

Scientific; catalog #: MA515307), and anti-beta-2-microglobulin (Cell Signaling Technology; catalog #: 59035S).

RNA-sequencing

The Eukaryote Total RNA Nano kit (Agilent Technologies; catalog #: 5067-1511) was used to check the quality of RNAs on an Agilent bioanalyzer. A total of 800ng RNA was then used for library preparation with the KAPA RNA Hyper+RiboErase HMR kit (Roche Diagnostics; Catalog #: 08098140702), following the instructions from the user manual. Briefly, the ribosomal RNA was removed by enzymatic digestion, and then the RNA was fragmented to around 200-300 bp with heat in fragmentation buffer. Synthesis of cDNA was conducted with reverse transcriptase and random primer, and then the second strand was synthesized to generate double-stranded cDNA. Following the repair of the DNA ends, NEB adapter was ligated, and the DNA are amplified with the KAPA HiFi HotStart mix and NEB dual barcode. Quality of the library was checked using the bioanalyzer with the Agilent DNA 1000 Kit (Agilent Technologies; catalog #: 5067-1504), and the sequencing was performed using NovaSeq 6000. The RNA-sequencing data was analyzed with packages *limma* (3, 4) and *edgeR* (5). Gene set enrichment analysis was conducted on the ranked gene set (log2 fold-change * -log10(p-value)) using the *fgsea* package. The analyzed hallmark pathways were collected from the Molecular Signatures Database (6, 7). Mouse-specific bulk deconvolution tools, seq-ImmuCC (8) and mMCP-counter (9), were used to estimate the differential relative abundance of $CD8^+$ T cells.

Analysis of single-cell RNA-sequencing in ICB-treated cohorts

Three publicly available single-cell RNA-sequencing datasets with clinical outcomes were employed to assess pre-treatment *PIKFYVE* expression. These datasets included Melanoma-derived brain metastases (MBM) (10), Melanoma (11), and Breast cancer (12). The raw count data for Melanoma and Breast cancer was downloaded from https://lambrechtslab.sites.vib.be/en webpage, while MBM data was download from Human Cell Atlas (https://www.humancellatlas.org/) website. Cell-type annotations were extracted from the original studies. Each dataset was subsetted for malignant cells in pre-treatment samples. Single-cell analysis was performed using the Seurat package (v4.1.1). Data normalization was performed using log1p normalization and clustering was undertaken using Seurat's unsupervised graph-based clustering approach. For evaluating *PIKFYVE* expression per patient,

FetchData function from Seurat was used employed to extract *PIKFYVE* expression from each cell and the result was aggregated on a patient level. For each study the patient level expression was scaled, and patients were stratified between *PIKFYVE* high and *PIKFYVE* low. The difference between the two categories for each study was evaluated using a chi-square test. The results were then combined from the three studies into a unified table and chi-square test was again performed to evaluate the differences between *PIKFYVE* level for favourable and unfavorable patient responses. Favourable response included responders and expanded patients while unfavourable responses were non-responders and non-expanded patients.

Cell proliferation assay

Proliferation of cancer cells *in vitro* was measured by the confluence of the cells on 96-well-dishes, using the IncuCyte ZOOM system.

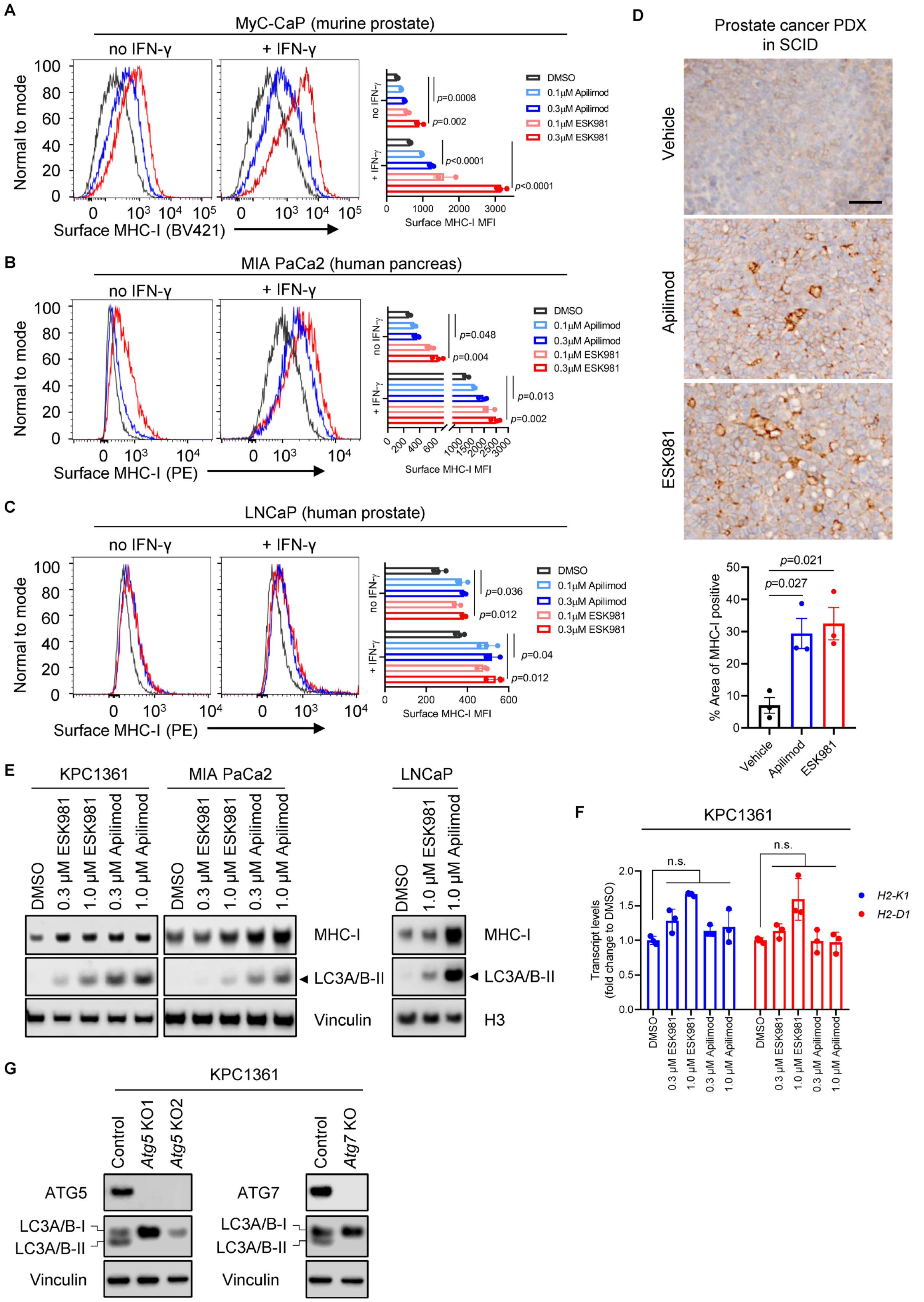

Fig. S1. (**A**-**C**) Representative images (left) and quantification (right) of flow cytometry measuring MHC-I surface expression in MyC-CaP (**A**), MIA PaCa2 (**B**), and LNCaP (**C**), stimulated with or without IFN-γ at 10 ng/ml, and treated with the agents at the indicated concentrations for 24 hours. (**D**)

Representative images (top) and quantification (bottom) of immunohistochemistry measuring MHC-I expression in the indicated prostate cancer patient-derived xenografts (PDX) in severe combined immunodeficiency (SCID) mice. Data was acquired from three biological replicates. The mice were treated with the indicated agent once daily at 30 mg/kg for five days. Scale bar: 50 μm. (**E**) Immunoblot analysis assessing levels of the indicated proteins in the indicated cells treated with the indicated agents for 24 hours. Images are representative of two independent biological replicates. (**F**) RT-qPCR measuring expression of the indicated genes in KPC1361 treated with the indicated agent for 24 hours. Data were acquired with technical triplicates. (**G**) Western blot assessing knockout efficacy on *Atg5* or *Atg7* in KPC1361.

Data in **A**, **B**, and **C** were acquired with three independent biological replicates. All data are presented as mean ± s.d. Statistics were acquired with Bonferroni correction by two-tailed Student's t-test (**A-D**), or by two-way ANOVA (**F**). MFI: mean fluorescence intensity. Control: non-targeting single-guide RNA. *Pikfyve* KO1 and *Pikfyve* KO2: independent single-guide RNAs depleting *Pikfyve*.

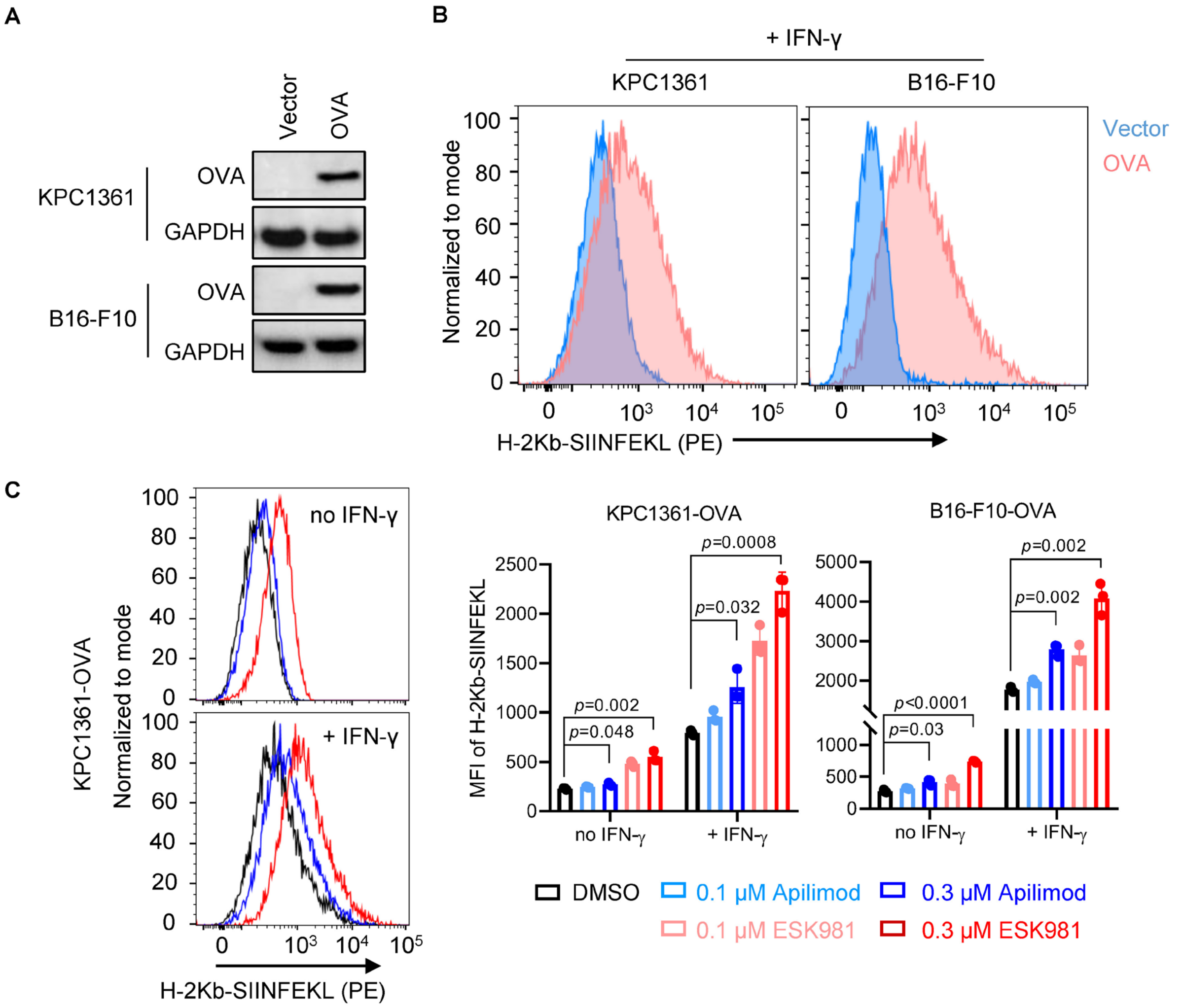

Fig. S2. (**A**) Western blot assessing expression of ovalbumin (OVA) in the indicated cells with or without OVA overexpression. (**B**) Representative images of flow cytometry measuring surface expression of an OVA-peptide (SIINFEKL) bound MHC-I (H-2Kb) in the indicated cells, treated with IFN-γ at 10 ng/ml for 24 hours. Images in **A** and **B** are representative of two independent biological replicates. (**C**) Representative images (left) and quantification (right) of flow cytometry measuring surface expression of an OVA-peptide (SIINFEKL) bound MHC-I (H-2Kb) in the indicated cells stimulated with or without IFN-γ at 10 ng/ml and treated with the indicated agents at the indicated concentrations for 24 hours.

Data in **C** were acquired with technical triplicates presented as mean ± s.d. Statistics were acquired by two-tailed Student's t-test, with Bonferroni correction. MFI: mean fluorescence intensity.

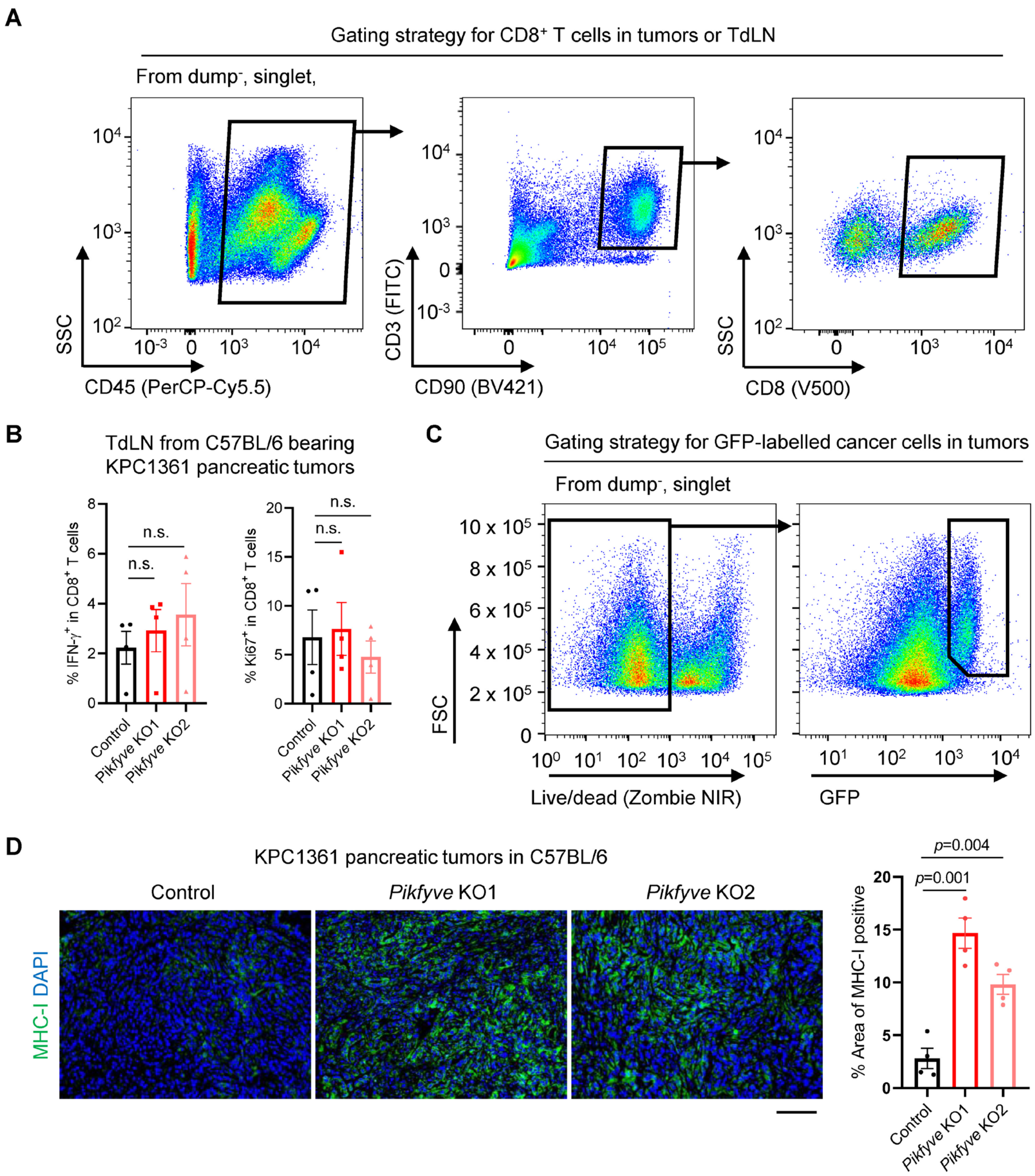

Fig. S3. (**A**) Gating strategy in flow cytometry identifying CD8$^+$ T cells. (**B**) Quantification of flow cytometry measuring the proportion of activated or proliferative CD8$^+$ T cells in the tumor-draining lymph nodes (TdLN) from C57BL/6 mice bearing the indicated KPC1361 pancreatic tumors (n = 4, per group). (**C**) Gating strategy in flow cytometry identifying GFP-labelled cancer cells in tumors. (**D**) Representative (left) and quantification (right) of MHC-I expression in pancreatic tumors (n = 4, per group) established with orthotopic injection of KPC1361 in C57BL/6 mice. Scale bar: 100 μm.

Data are presented as mean ± s.e.m. Statistics were acquired by two-tailed Student's t-test, with Bonferroni correction. Control: non-targeting single-guide RNA. *Pikfyve* KO1 and *Pikfyve* KO2: independent single-guide RNAs depleting *Pikfyve*.

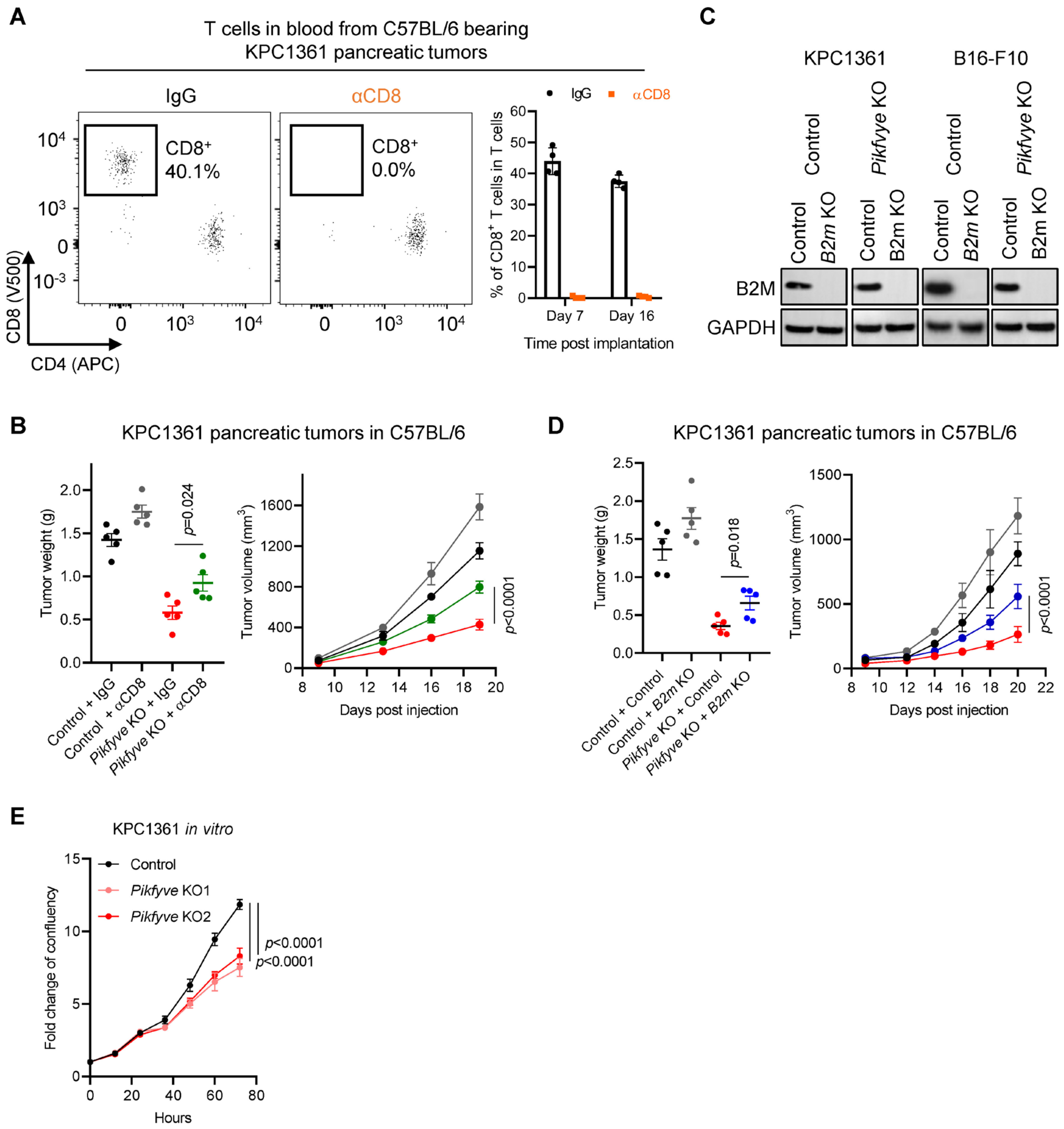

Fig. S4. (**A**) Representative images (left) and quantification (right) of flow cytometry measuring the amount of CD8$^+$ T cells in the blood harvested on the indicated time from tumor-bearing C57BL/6 mice treated with isotype control (IgG) or anti-mouse CD8 antibody (αCD8) at 400 µg/mouse on day 2 post cancer cell implantation, and subsequently at 100 µg/mouse once every three days. (**B**) Weights (left) and volumes (right) of pancreatic tumors established with orthotopic injection of control or *Pikfyve*-null KPC1361 cells to C57BL/6 mice, with or without depletion of CD8$^+$ T cells as in **A** (n = 5, per group). Data were reproducible with independent *Pikfyve*-knockout single-guide RNA. (**C**) Western blot assessing expression of Beta-2 microglobulin (B2M) after *B2m* depletion by single-guide RNA (*B2m* KO) in the indicated cells. (**D**) Weights (left) and volumes (right) of pancreatic tumors established with orthotopic injection of control, *B2m* or *Pikfyve* single knockout, or *B2m* and *Pikfyve* double knockout KPC1361 cells to C57BL/6 (n = 5, per group). Data were reproducible with independent *B2m*-knockout single-guide RNA. (**E**) Confluency of the indicated cells over the indicated time. Data were acquired with technical triplicates.

All data are presented as mean ± s.d. in **A** and **E**, and as mean ± s.e.m. in **B** and **D**. Statistics were acquired by two-tailed Student's t-test in tumor weight data in **B** and **D**, or by two-way ANOVA in tumor volume in **B**, **D**, and in **E**. Control: non-targeting single-guide RNA.

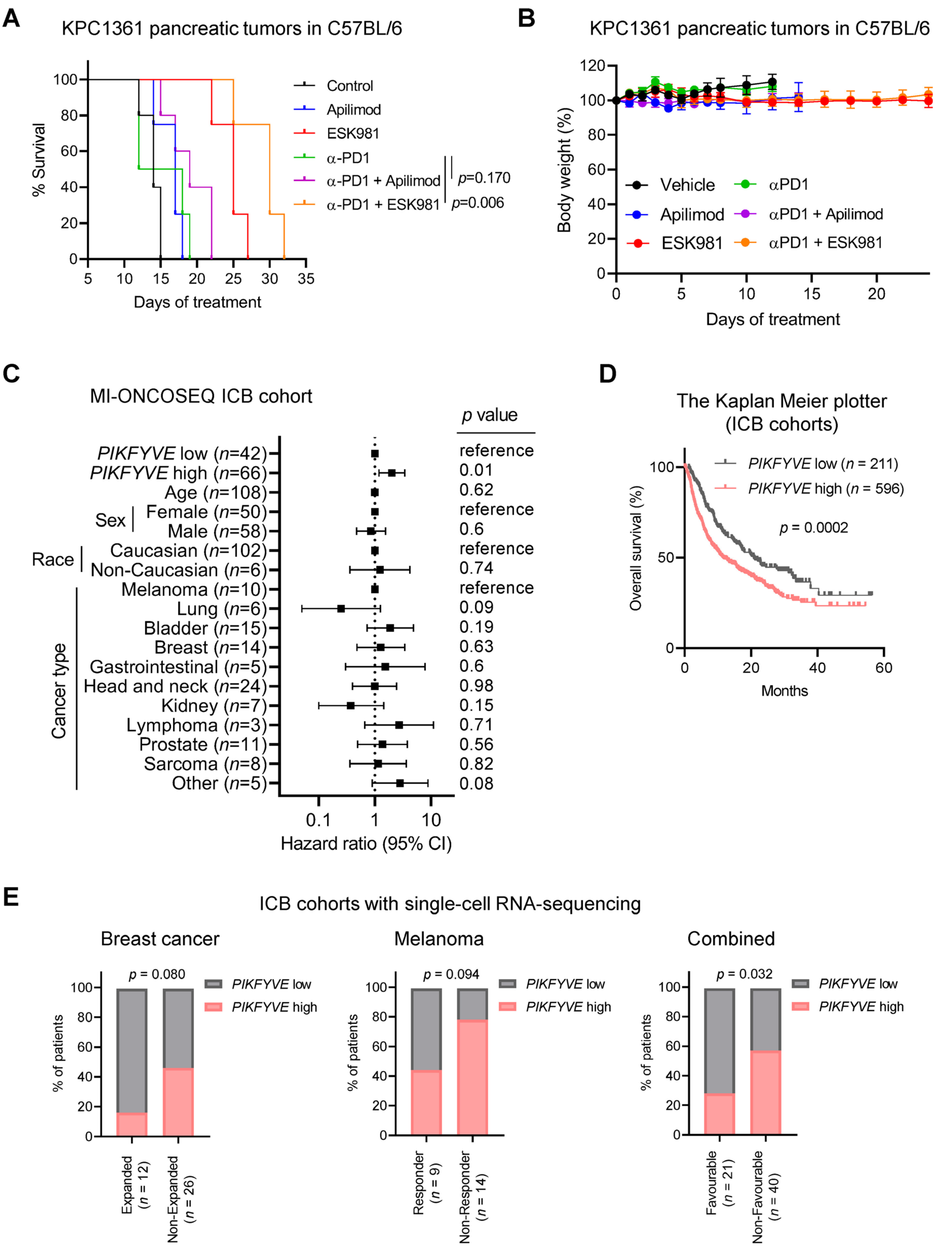

Fig. S5. (**A**) Survival curves of C57BL/6 mice bearing pancreatic tumors established with orthotopic injection of KPC1361 cells and treated with the indicated agents. *N* = 4 mice per group, except for the apilimod-treated (*n* = 5 mice, per group). ESK981 or apilimod were administrated once daily at 30 mg/kg or 60 mg/kg, respectively. Anti-PD1 (α-PD1) was administrated biweekly at 250 µg/mouse. Statistics were acquired by log-rank test. (**B**) Body weight of the mice in **A**, along treatment. Data are presented as mean ± s.d. (**C**) Cox regression analysis for overall survival on pre-treatment *PIKFYVE* expression (high, *n* = 66, versus low, *n* = 42) and the indicated variables in MI-ONCOSEQ ICB cohort (*n* = 108). CI: confidence interval. (**D**) Overall survival of patients with tumors showing high or low pretreatment

PIKFYVE mRNA levels in ICB-treated cohorts. Data was acquired from the Kaplan Meier plotter (https://kmplot.com/analysis/). Statistics was acquired by log-rank test. (**E**) Analysis of single-cell RNA-sequencing for the association between pre-treatment *PIKFYVE* expression in malignant cells and clinical outcomes in ICB-treated cohorts. Left: Expanded patients (patients showing T cell-expansion after ICB treatment) in a breast cancer cohort. Middle: Association between pre-treatment *PIKFYVE* expression in malignant cells and response to ICB in melanoma cohorts. Right: Combination of the left and middle. Statistics were acquired by chi-square tests.

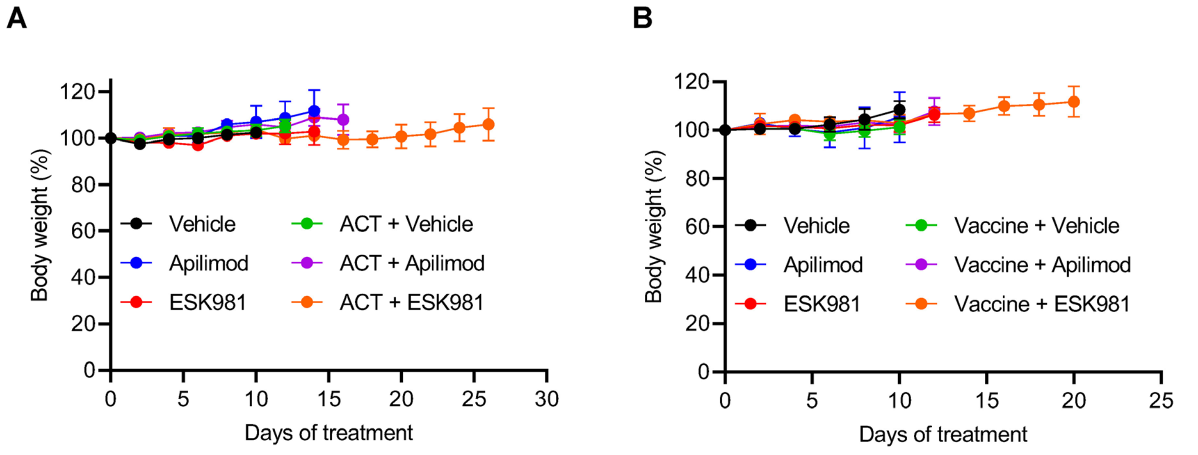

Fig. S6. (**A** and **B**) Body weight of mice treated with ACT or therapeutic vaccine in combination with apilimod or ESK981. Data are presented as mean ± s.d.

Primer	Sequence
*Actb*_ forward	GGCCAACCGTGAAAAGATGA
Actb _reverse	TACGACCAGAGGCATACAGG
H2-K1 _forward	CCCTGTGAGCCTATGGACTC
H2-K1 _reverse	TGTGGAAGGGAAGACAGAGC
H2-D1 _forward	ACATCCAGAGCCCTCAGTTC
H2-D1 _reverse	GGCTCCACAGTTCTTCACAC

Table S1. Sequences of RT-qPCR primers used in this study.

sgRNA	Target sequence
sg*Pikfyve* #1	CTAACACTGAAGAGCGCCGG
sg*Pikfyve* #2	TGACAAGAGTTCCCCGACAC
sg*Atg5* #1	GGCCATCAACCGGAAACTCA
sg*Atg5* #2	TCCATCCAAGGATGCGGTTG
sgAtg7	GAAGTTGAACGAGTACCGCC
sg*B2m* #1	ATTTGGATTTCAATGTGAGG
sg*B2m* #2	AGTATACTCACGCCACCCAC
Nontargeting control	ACGTGTAAGGCGAACGCCTT

Table S2. Target sequences of sgRNAs used in this study.

Data S1. (Excel file) Clinical data and *PIKFYVE* expression of MI-ONCOSEQ ICB cohort.

References

1. K. Yamamoto *et al.*, Autophagy promotes immune evasion of pancreatic cancer by degrading MHC-I. *Nature* **581**, 100-105 (2020).
2. K. Koikawa *et al.*, Targeting Pin1 renders pancreatic cancer eradicable by synergizing with immunochemotherapy. *Cell* **184**, 4753-4771 e4727 (2021).
3. M. E. Ritchie *et al.*, limma powers differential expression analyses for RNA-sequencing and microarray studies. *Nucleic Acids Res* **43**, e47 (2015).
4. B. Phipson, S. Lee, I. J. Majewski, W. S. Alexander, G. K. Smyth, Robust Hyperparameter Estimation Protects against Hypervariable Genes and Improves Power to Detect Differential Expression. *Ann Appl Stat* **10**, 946-963 (2016).
5. M. D. Robinson, D. J. McCarthy, G. K. Smyth, edgeR: a Bioconductor package for differential expression analysis of digital gene expression data. *Bioinformatics* **26**, 139-140 (2010).
6. A. Subramanian *et al.*, Gene set enrichment analysis: a knowledge-based approach for interpreting genome-wide expression profiles. *Proc Natl Acad Sci U S A* **102**, 15545-15550 (2005).
7. A. Liberzon *et al.*, The Molecular Signatures Database (MSigDB) hallmark gene set collection. *Cell Syst* **1**, 417-425 (2015).
8. Z. Chen *et al.*, seq-ImmuCC: Cell-Centric View of Tissue Transcriptome Measuring Cellular Compositions of Immune Microenvironment From Mouse RNA-Seq Data. *Front Immunol* **9**, 1286 (2018).
9. F. Petitprez *et al.*, The murine Microenvironment Cell Population counter method to estimate abundance of tissue-infiltrating immune and stromal cell populations in murine samples using gene expression. *Genome Med* **12**, 86 (2020).
10. C. Alvarez-Breckenridge *et al.*, Microenvironmental Landscape of Human Melanoma Brain Metastases in Response to Immune Checkpoint Inhibition. *Cancer Immunol Res* **10**, 996-1012 (2022).
11. J. Pozniak *et al.*, A TCF4/BRD4-dependent regulatory network confers cross-resistance to targeted and immune checkpoint therapy in melanoma. *bioRxiv* 10.1101/2022.08.11.502598, 2022.2008.2011.502598 (2022).
12. A. Bassez *et al.*, A single-cell map of intratumoral changes during anti-PD1 treatment of patients with breast cancer. *Nature Medicine* **27**, 820-+ (2021).
